# Cardioprotective effects of curcumin against myocardial I/R injury: A systematic review and meta-analysis of preclinical and clinical studies

**DOI:** 10.3389/fphar.2023.1111459

**Published:** 2023-03-09

**Authors:** Tianli Li, Jialin Jin, Fenglan Pu, Ying Bai, Yajun Chen, Yan Li, Xian Wang

**Affiliations:** ^1^ Department of Cardiology, Dongzhimen Hospital, Beijing University of Chinese Medicine, Beijing, China; ^2^ National Integrated Traditional and Western Medicine Center for Cardiovascular Disease, China-Japan Friendship Hospital, Beijing, China; ^3^ Center for Evidence Based Chinese Medicine, Beijing University of Chinese Medicine, Beijing, China; ^4^ Department of Traditional Chinese Medicine, Peking Union Medical College Hospital, Beijing, China; ^5^ Department of Cardiology, Dongfang Hospital, Beijing University of Chinese Medicine, Beijing, China

**Keywords:** curcumin, myocardial ischemia-reperfusion injury (MIRI), systematic review and meta-analysis, pre-clinical evidence, clinical evidence

## Abstract

**Objective:** Myocardial ischemia-reperfusion (I/R) injury is a complex clinical problem that often leads to further myocardial injury. Curcumin is the main component of turmeric, which has been proved to have many cardioprotective effects. However, the cardioprotective potential of curcumin remains unclear. The present systematic review and meta-analysis aimed to evaluate the clinical and preclinical (animal model) evidence regarding the effect of curcumin on myocardial I/R injury.

**Methods:** Eight databases and three register systems were searched from inception to 1 November 2022. Data extraction, study quality assessment, data analyses were carried out strictly. Then a fixed or random-effects model was applied to analyze the outcomes. SYRCLE’s-RoB tool and RoB-2 tool was used to assess the methodological quality of the included studies. RevMan 5.4 software and stata 15.1 software were used for statistical analysis.

**Results:** 24 animal studies, with a total of 503 animals, and four human studies, with a total of 435 patients, were included in this study. The meta-analysis of animal studies demonstrated that compared with the control group, curcumin significantly reduced myocardial infarction size (*p* < 0.00001), and improved the cardiac function indexes (LVEF, LVFS, LVEDd, and LVESd) (*p* < 0.01). In addition, the indexes of myocardial injury markers, myocardial oxidation, myocardial apoptosis, inflammation, and other mechanism indicators also showed the beneficial effect of curcumin (*p* < 0.05). In terms of clinical studies, curcumin reduced the incidence of cardiac dysfunction, myocardial infarction in the hospital and MACE in the short term, which might be related to its anti-inflammatory and anti-oxidative property. Dose-response meta-analysis predicted, 200 mg/kg/d bodyweight was the optimal dose of curcumin in the range of 10–200 mg/kg/d, which was safe and non-toxic according to the existing publications.

**Conclusion:** Our study is the first meta-analysis that includes both preclinical and clinical researches. We suggested that curcumin might play a cardioprotective role in acute myocardial infarction in animal studies, mainly through anti-oxidative, anti-inflammatory, anti-apoptosis, and anti-fibrosis effects. In addition, from the clinical studies, we found that curcumin might need a longer course of treatment and a larger dose to protect the myocardium, and its efficacy is mainly reflected on reducing the incidence of myocardial infarction and MACE. Our finding provides some meaningful advice for the further research.

## 1 Introduction

Cardiovascular disease (CVD), especially acute myocardial infarction (AMI), has become one of the main risk factors threatening human health (Mozaffarian et al., 2015). Thrombolytic, percutaneous coronary intervention (PCI), and coronary artery bypass grafting (CABG) are currently effective strategies to limit infarct size and reduce mortality in clinical practice (Ribas et al., 2017). However, there is growing evidence indicating that myocardial ischemia/reperfusion (I/R) injury is an inevitable pathological change in the process of reperfusion after revascularization (Heusch and Gersh, 2017). In addition, it will lead to myocardial dysfunction, structural damage, and myocardial electrical activity disorder, which further aggravates myocardial necrosis, severe complications such as arrhythmia, decreased ventricular function, and even sudden death (Grech et al., 1995). The mechanism is not yet fully understood, but research showed that it might be related to inflammation, oxidative stress, mitochondrial membrane permeability transition pore, cell apoptosis, et al. (Hausenloy and Yellon, 2013). Therefore, seeking novel myocardial protection strategies to ameliorate myocardial I/R injury and preserve normal heart function after revascularization is still of high priority.

**FIGURE 1:**
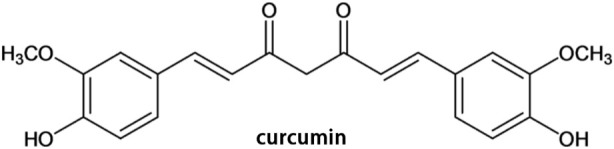
Chemical structure of curcumin.

Turmeric is a commonly used herb as a traditional medicine in Asian countries (China, Japan, Korea, et al.). According to the records of ancient Chinese medical books, turmeric is capable of relieving pain through promoting qi and blood circulation. Therefore, it is frequently used in the treatment of chest tightness, chest pain, shoulder and back pain, dysmenorrhea, et al. Curcumin (chemical structure shown in Figure 1) is the main component of turmeric and has a variety of biological activities, especially in the cardiovascular system (Ghosh et al., 2010). Curcumin has been proved to have anti-oxidative, anti-inflammatory, and anti-apoptosis properties, and it has been observed in experiments that curcumin inhibits myocardial fibrosis (Gorabi et al., 2020) and ventricular remodeling after acute infarction (Yao et al., 2004) and prevents the progression of heart failure after myocardial infarction (Saeidinia et al., 2018). Therefore, curcumin may be a potential cardioprotective candidate to ameliorate myocardial I/R injury.

However, the efficacy and mechanisms of curcumin on myocardial I/R injury have not been systematically reviewed in animal models. Moreover, some clinical trials (Offermanns et al., 1994; Srivastava et al., 1995; Peng and Qian, 2014; Hirsch et al., 2017) showed the contradictory results. In this study, we aim to review and analyze the animal and clinical research to find out whether curcumin has the potential for clinical application and suggested how such primary studies need to be improved.

## 2 Methods

### 2.1 Search strategies and study selection

Eight databases, including Pubmed, EMBASE, The Cochrane Library, Web of Science, China National Knowledge Infrastructure (CNKI), Wanfang database, China Science and Technology Journal Database (VIP), and China Biology Medicine disc (CBM), were searched systematically from inception to 1 November 2022. Moreover, references of eligible studies were also manually searched to identify additional eligible studies. The search language was restricted to English and Chinese, and the search strategy was as follows. The detailed search strategies in eight databases and retrieved results were provided in Supplementary Material.

### 2.2 Eligibility criteria

Inclusion criteria were as follows: 1) The I/R experimental model were established by ligating the left coronary artery (LCA) such as left anterior descending (LAD) or other proper methods, or ischemic heart disease patients receiving PCI or CABG in the hospital; 2) The treatment group (animal studies and human studies) received any dose of curcumin as monotherapy, and the control group received the same amount of non-functional fluid (normal saline) or no treatment at all; 3) The primary outcome of animal studies were myocardial infarction (MI) size and echocardiogram indicators. The secondary outcomes of animal studies were biomarkers of myocardial injury and mechanisms of curcumin in the treatment of myocardial I/R injury; 4) The human studies were RCT study. Exclusion criteria were set as follows: 1) not a myocardial I/R model or patients who beyond the relevant clinical diagnostic criteria; 2) combined with other drugs; 3) no control group; 4) duplicate publication; 5) not an animal model or not RCT study (e.g., case reports, reviews, non-RCT clinical trials and cell experiments).

### 2.3 Data extraction

Two independent reviewers (Fenglan Pu, Ying Bai) extracted the following details from the included studies: 1) first author name and year of publication; 2) Specific information about the animals and patients in each study, including species, number, sex, and body weight; 3) Myocardial I/R model and the anesthetic method used to prepare the model; 4) Information about curcumin treatment, including dose, method of administration, course of treatment; as well as corresponding information in the control group; 5) The mean and standard deviation (SD) of the results. Suppose there were many different time point results, only the last time point results would be recorded. Since some of the data is only presented in graphical format, we tried to contact the authors for detailed information. If we did not receive any response, the digital ruler software would be used to measure the value of the graph.

### 2.4 Risk of bias in individual studies

The quality of the included animal studies was assessed using Systematic Review Center for Laboratory Animal Experimentation (SYRCLE) risk of bias (RoB) tool 10-item scale (Hooijmans et al., 2014) as follows (A) sequence generation; (B) baseline characteristics; (C) allocation concealment; (D) random housing; (E) blinding investigators; (F) random outcome assessment; (G) blinding outcome assessor; (H) incomplete outcome data; (I) selective outcome reporting; (J) other sources of bias. Give one point to each item.

The quality of the included human studies was assessed using RoB-2 tool (Sterne et al., 2019) as follows: Randomization process; Assignment to intervention; Adhering to intervention; Missing outcome data; Measurement of outcome; Selection of the reported result; RoB-2 overall score. Two reviewers (Jialin Jin and Fenglan Pu) assessed the study quality independently. The divergences were resolved through negotiation or in consultation with the corresponding author (Yan Li and Xian Wang).

### 2.5 Data synthesis

The problem of curcumin divided into different dose subgroups in the original study was handled following the strategy recommended by Cochrane Handbook for Systematic Reviews of Interventions (CHSRI) (Cumpston et al., 2019). We merged the different dose subgroups in each original study into one treatment group, and the following was the merge formula for continuous variable (Zhou et al., 2020):

$$Sample\;size\;N_{1}+N_{2}+...+N_{k}$$

$$Mean\;\frac{N_{1}M_{1}+N_{2}M_{2}+...+N_{k}M_{k}}{N_{1}+N_{2}+...+N_{k}}$$

$$SD\;\sqrt{\frac{\left(N_{1}-1\right)SD_{1}^{2}+\left(N_{2}-1\right)SD_{2}^{2}+...+\left(N_{k}-1\right)SD_{K}^{2}}{N_{1}+N_{2}+...+N_{k}-k}}$$

### 2.6 Statistical analysis

RevMan software (version 5.4) was used for statistical analysis. Heterogeneity was determined by the Q-statistical test (*p* < 0.05 was considered statistically significant) and I^2^-statistical test. According to the Cochrane handbook on heterogeneity analysis, I^2^ value ranges from 0% to 40% means heterogeneity might not be important; 30%–60% may represent moderate heterogeneity; 50%–90% may represent substantial heterogeneity; 75%–100% means considerable heterogeneity. A fixed-effects model was adopted if I^2^ < 50%; otherwise, a random-effects model would be applied. All outcomes were continuous variables, so we used weighted mean difference (WMD) with 95% confidence intervals (CIs) to present, for outcomes reported in various measurement methods or different scales of measurement, we calculated with a standard mean difference (SMD). *p* < 0.05 was considered statistically significant.

### 2.7 Subgroup analysis and meta-regression

We preset four subgroups: 1) method of model establishment, 2) administration method, 3) genus of animals, 4) intervention moment. We conducted a subgroup analysis on the primary outcomes to evaluate the impact of variables and to explore the source of heterogeneity. A meta-regression analysis was further performed using Stata 15.1 software once the heterogeneity in the study was significant (I^2^ > 50%) and the preset subgroups failed to find the source of heterogeneity.

### 2.8 Sensitivity analysis

Sensitivity analysis should be performed on the outcomes whose heterogeneities were considerable. The purpose of the sensitivity analysis was not only to indirectly find the source of heterogeneity, but also to assess the stability and reliability of the merged results.

### 2.9 Publication bias

Publication bias was detected by a funnel plot combined with Egger’s regression test on primary outcomes. The funnel plot was produced using RevMan 5.4 software, and Egger’s regression test was completed using Stata 15.1 software.

### 2.10 Dose-response meta-analysis

The relationship between the standard dosage of curcumin and the ratio of means (RR = means_experimental_/means_control_) was modeled using STATA 15.1 software. According to the characteristics of point distribution, linear, exponential, logarithmic, quadratic, and cubic regression equations can be selected to try to fit. The generalized least squares method (GLS) was used to calculate each of the parameters while *R* ^2^ represents the degree of fitting (*R* ^2^ closer to one indicates a better fit of the prediction model).

## 3 Results

### 3.1 Study selection

**FIGURE 2:**
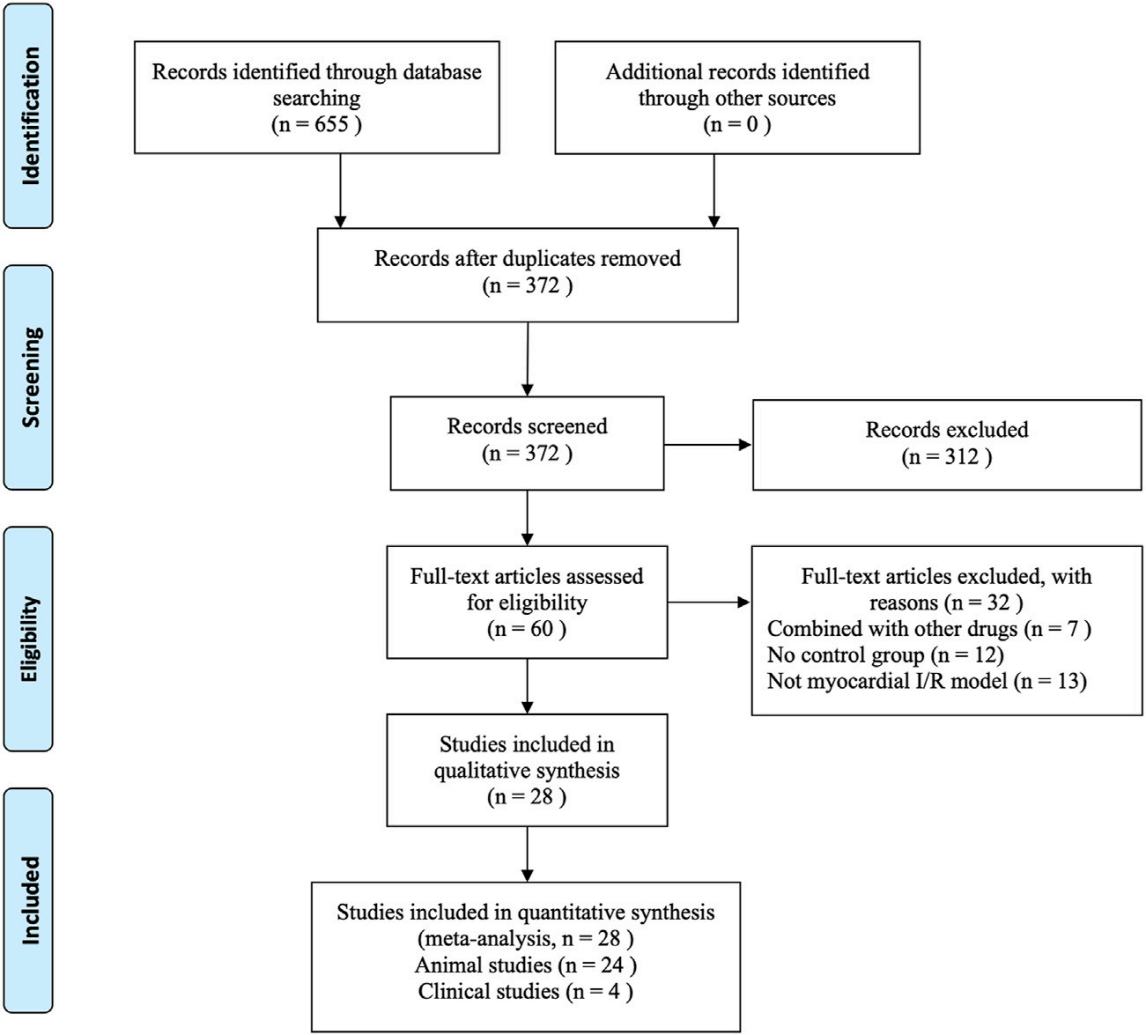
Flow chart of the process of study selection.

A total of 655 articles were retrieved from the eight databases. After the removal of duplicates using Endnote X9 software, 372 articles were screened through reading titles and abstracts by two reviewers individually (Jialin Jin and Fenglan Pu). Then the same two reviewers (Jialin Jin and Fenglan Pu) individually perused the remaining 60 full-texts, and finally 24 animal studies (Yeh et al., 2005; Ali et al., 2009; Gonzalez-Salazar et al., 2011; Duan et al., 2012; Jeong et al., 2012; Kim et al., 2012; Wang et al., 2012; Broskova et al., 2013; Wang et al., 2014; Yao and Jiang, 2014; Hardy et al., 2015; Chen et al., 2016; Liu et al., 2017a; Deng et al., 2018; Wang et al., 2018) published from 2005 to 2020 and four human studies (Wongcharoen et al., 2011; Wongcharoen et al., 2012; Aslanabadi et al., 2019; Phrommintikul et al., 2019) published from 2011 to 2019 were included in our systematic review and meta-analysis. During the selection process, any divergence was resolved by Ying Bai. Figure 2 describes the flow chart for inclusion in the study.

### 3.2 Characteristics of included studies

#### 3.2.1 Animal studies

Twenty studies (Sato et al., 2000; Yeh et al., 2005; Kim et al., 2008; Ali et al., 2009; Gonzalez-Salazar et al., 2011; Duan et al., 2012; Jeong et al., 2012; Kim et al., 2012; Wang et al., 2012; Yang et al., 2013a; Broskova et al., 2013; Leong et al., 2013; Wang et al., 2014; Hardy et al., 2015; Chen et al., 2016; Liu et al., 2017a; Liu et al., 2017b; Deng et al., 2018; Wang et al., 2018; Jo et al., 2020) were published in English, and four studies (Cheng et al., 2005; Wu, 2011; Yao and Jiang, 2014; Gu et al., 2016) were published in Chinese. Sixteen studies (Sato et al., 2000; Cheng et al., 2005; Kim et al., 2008; Wu, 2011; Duan et al., 2012; Jeong et al., 2012; Kim et al., 2012; Wang et al., 2012; Leong et al., 2013; Wang et al., 2014; Liu et al., 2017a; Liu et al., 2017b; Wang et al., 2018; Jo et al., 2020) used Sprague-Dawley (SD) rats, five studies (Ali et al., 2009; Gonzalez-Salazar et al., 2011; Broskova et al., 2013; Hardy et al., 2015; Chen et al., 2016) used Wistar rats, two studies (Gu et al., 2016; Deng et al., 2018) used C57BL/6 mice, and one study (Yeh et al., 2005) used New Zealand white rabbits. Twenty studies (Cheng et al., 2005; Yeh et al., 2005; Kim et al., 2008; Ali et al., 2009; Gonzalez-Salazar et al., 2011; Wu, 2011; Duan et al., 2012; Jeong et al., 2012; Kim et al., 2012; Wang et al., 2012; Yang et al., 2013a; Broskova et al., 2013; Wang et al., 2014; Yao and Jiang, 2014; Hardy et al., 2015; Chen et al., 2016; Gu et al., 2016; Deng et al., 2018; Wang et al., 2018; Jo et al., 2020) used male animals, and the remaining four studies (Sato et al., 2000; Leong et al., 2013; Liu et al., 2017a; Liu et al., 2017b) did not clarify gender. Twelve studies (Sato et al., 2000; Cheng et al., 2005; Yeh et al., 2005; Gonzalez-Salazar et al., 2011; Wu, 2011; Duan et al., 2012; Jeong et al., 2012; Yang et al., 2013a; Leong et al., 2013; Yao and Jiang, 2014; Gu et al., 2016; Wang et al., 2018) used pentobarbital sodium for anesthetic, six studies (Kim et al., 2008; Kim et al., 2012; Wang et al., 2012; Wang et al., 2014; Liu et al., 2017b; Deng et al., 2018) used ketamine combined with xylazine, two studies (Chen et al., 2016; Liu et al., 2017a) used chloral hydrate, one study (Broskova et al., 2013) used diethylether, one study (Ali et al., 2009) used thiopentone sodium, one study (Jo et al., 2020) used Alfaxalone combined with xylazine, and the remaining one (Hardy et al., 2015) did not specify the anaesthetic. 15 myocardial I/R models (Cheng et al., 2005; Kim et al., 2008; Ali et al., 2009; Wu, 2011; Jeong et al., 2012; Kim et al., 2012; Wang et al., 2012; Yang et al., 2013a; Wang et al., 2014; Yao and Jiang, 2014; Chen et al., 2016; Gu et al., 2016; Liu et al., 2017b; Deng et al., 2018; Jo et al., 2020) were produced by ligation of the coronary artery, the rest were produced by turning off and then turning on the Langendorff heart perfusion system which is supposed to pump blood to the heart. The outcomes mentioned were presented as follows: myocardial infarction size, left ventricular ejection fractions (LVEF), left ventricular fraction shortening (LVFS), left ventricular end diastolic dimension (LVDd), left ventricular end systolic dimension (LVSd), lactate dehydrogenase (LDH), creatine kinase (CK), CK-MB, superoxide dismutase (SOD), catalase (CAT), glutathione (GSH), malondialdehyde (MDA), myeloperoxidase (MPO), nuclear factor kappa B (NF-κB), tumor necrosis factor alpha (TNF-a), cardiac fibrosis area. The detailed characteristics of the included studies are shown in Table 1.

**TABLE 1:** Basic characteristics of the 24 included animal studies.

Study ID	Country	Species (sex, n = experimental/control group)	Weight	Model (method)	Anesthetic	Treatment group (method to curcumin)	Control group	Duration	Outcomes	*p*-Value
Ali, M. S.2009	India	Albino Wistar rats (male, n = 12/6)	200–250 g	Block LAD for 30min then reperfuse for 4 h	Thiopentone sodium (30 mg/kg)	Curcumin (5/10 mg/kg) dissolved in 80% DMSO and given as an i.p. Injection	0.2 mL of 80% DMSO and given as an i.p. Injection	10 min before reperfusion	1.ALT	1.*p* < 0.05
									2.AST	2.*p* < 0.001
									3.Catalase	3.*p* < 0.05
									4.GSH	4.*p* < 0.001
									5.MDA	5.*p* < 0.05
Brosková.Z.2013	Slovak Republic	Wistar rats (male, n = 6/12)	360–400 g	Turn off pumpig system for 30min then reflow 30min using Langendorff technique	Diethylether	Curcumin dissolved in DMSO at a concentration of 1*10–5 mol/L then reperfused for 30 min	DMSO reperfused for 30 min	30 min during reperfusion	1.VPB	1.*p* < 0.05
									2.VTP	2.*p* < 0.05
									3.VFP	3.*p* < 0.05
Chen, X. L. 2016	China	albino Wistar strain rats (male, n = 3/3)	160–180 g	Block coronary artery for 45min then reperfuse	5% chloral hydrate solution	Curcumin (50 mg/kg bwt) oral	Saline oral	15 days before ischemia	1.Cat	1.*p* < 0.05
									2.Caspase-3 protein	2.*p* < 0.05
									3.Cell viability%	3.*p* < 0.05
									4.CKMB	4.*p* < 0.05
									5.IA/LV	5.*p* < 0.05
									6.ISR%	6.*p* < 0.05
									7.MA	7.*p* < 0.05
									8.MDA	8.*p* < 0.05
									9.SOD activity	9.*p* < 0.05
Cheng, H.2005	China	Sprague-Dawley rats (male, 60/20)	270 ± 20 g	Block LAD for 60 min then reflow for 60 min	Pentobarbital sodium (30 mg/kg, 3%)	Intraperitoneal injection curcumin (10/20/40 mg/kg)	Intraperitoneal injection saline 5 min	5 min earlier before ischemia	1.AR/LV	1.*p* < 0.05
									2.CK	2.*p* < 0.05
									3.GSH Px	3.*p* < 0.05
									4.IR/AR	4.*p* < 0.05
									5.IR/LV	5.*p* < 0.05
									6.LDH	6.*p* < 0.05
									7.MDA	7.*p* < 0.05
									8.SOD	8.*p* < 0.05
Deng Y.2018	China	C57BL/6 mice (male, n = 10/10)	20–25 g	Block LCA for 30 min then reperfuse for 4 h	Ketamine (100 mg/kg) and xylazine (5 mg/kg)	25ul,1 wt% curcumin solution into ischemia region with a 30-gauge needle	25ul,1 wt% normal saline into ischemia region with a 30-gauge needle	During reperfusion	1. Apoptosis	1.*p* < 0.01
									2.AR/LV	2.*p* < 0.05
									3.IR/AR	3.*p* < 0.05
									4.LVEF	4.*p* > 0.05
									5.LVFS	5.*p* > 0.05
									6.Bcl-2	6.*p* < 0.05
Duan, W.2012	China	Sprague-Dawley rats (male, n = 8/8)	220–250 g	Turn off pumpig system for 60 min then reflow 60 min using Langendorff technique	Pentobarbital sodium (50 mg/kg)	Perfused with KHB containing 1-lM curcumin	Perfused with KHB	During reperfusion for 10 min	1.Apoptosis	1.*p* < 0.05
									2.Bcl-2	2.*p* < 0.05
									3.DP/dtmax	3.*p* < 0.05
									4.HR	4.*p* < 0.05
									5.IR/LV	5.*p* < 0.05
									6.LDH	6.*p* < 0.05
									7.LVDP	7.*p* < 0.05
									8.p-STAT3	8.*p* < 0.05
González-Salazar, A.2011	Mexico	Wistar rats (male, n = 6/6)	270–300 g	Turn off pumpig system for 30min then reflow 60min using Langendorff technique	Pentobarbital sodium (60 mg/kg)	Curcumin (200 mg/kg) dissolved in carboxymethylcellulose 0.05% oral	Carboxymethylcellulose 0.05% oral	7 days before ischemia	1.GSH	1.*p* < 0.05
									2.SOD	2.*p* < 0.05
									3.CAT	3.*p* < 0.05
									4.OC	4.*p* < 0.05
Gu, H.P.2016	Chinese	C57BL/6 mice (male, n = 10/10)	18–22 g	Block LAD for 30 min then reflow for 4 h	Pentobarbital sodium (50 mg/kg)	By intraperitoneal injection of curcumin (100 mg/kg) 30 min	By intraperitoneal injection of saline 30 min	Before ischemia	1.IR/LV	1.*p* < 0.001
									2.cTnT	2.*p* < 0.001
									3.NF-κB protein	3.*p* < 0.001
									4.TNF-α protein	4.*p* < 0.001
Hardy, N.2015	Australian	Wistar rats (male, n = 5/4)	NM	Turn off pumpig system for 30min then reflow 60min using Langendorff technique	NM	Curcumin (0.177 mg/mL) and nanoparticle (1 mg/mL) were pumped through the column at the flow rate of 1.5 mL/min with a run time of 10 min for 3 times	Nanoparticle (1 mg/mL) was pumped through the column at the flow rate of 1.5 mL/min with a run time of 10 min for 3 times	After reperfusion	1.CK	1.*p* < 0.05
									2.LDH	2.*p* < 0.05
									3.GGR	3.*p* < 0.05
Jeong, C.W.2012	Korea	Sprague-Dawley rats (male, n = 7/7)	250–300 g	Block LAD for 30 min then reflow for 120 min	Pentobarbital sodium (60 mg/kg)	Curcumin (100 mg/kg/day) oral	Saline oral	20 min before ischemia	1.AR/LV	1.*p* < 0.05
									2.HR	2.*p* < 0.05
									3.MAP	3.*p* < 0.05
									4.p-AKT	4.*p* < 0.05
Jo, W.2020	South Korea	Sprague-Dawley rats (male, 5/5)	285.33 ± 5.0 g	Block LAD for 30 min then reflow	Alfaxalone (50 mg/kg) and xylazine (5 mg/kg)	Curcumin gavage (25 mg/kg/d)	Vehicle (10% dimethyl sulfoxide and 90% polyethylene glycol) gavage	5 days before ischemia	1. IR/LV	1.*p* < 0.01
									2.LVEF	2.*p* < 0.05
									3.LVFS	3.*p* < 0.05
									4.LVDd	4.*p* > 0.05
									5.LVSd	5.*p* > 0.05
									6.SV	6.*p* > 0.05
									7.Fibrosis/LV	7.*p* < 0.05
Kim, Y.S.2008	Korea	Sprague-Dawley rats (male, n = 10/10)	190–210 g	Block LAD for 30 min then reflow for 24 h	Ketamine (1 mL/kg) and xylazine (10 mg/kg)	By intragastric gavage of curcumin (80 mg/kg/d)	By intragastric gavage of saline	7 days before ischemia	1.Fibrosis/LV	1.*p* < 0.05
									2.NOAC	2.*p* < 0.05
									3.LVDd	3.*p* > 0.05
									4.LVSd	4.*P*0.05
									5.LVFS	5.*p* < 0.05
									6.MPO	6.*p <* 0.05
									7.MDA	7.*p* < 0.05
Kim, Y.S.2012	Korea	Sprague-Dawley rats (male, n = 6/8)	200–230 g	Block LAD for 30 min then reflow for 2 weeks	Ketamine (50 mg/kg) and xylazine (5 mg/kg)	Curcumin (300 mg/kg/day) dissolved in distilled water oral	Distilled water oral	7 days befor ischemia and 14 days during reperfusion	1.Fibrosis/LV	1.*p* < 0.05
									2.LVFS	2.*p* < 0.05
									3.LVEF	3.*p* < 0.05
									4.LVDd	4.*p* < 0.05
									5.LVSd	5.*p* < 0.05
									6.Max dp/dt	6.*p* < 0.05
									7.Min dp/dt"	7.*p* < 0.05
Leong, P.K.2013	China (Hong Kong)	Sprague-Dawley rats (NM, n = 5/5)	250–300 g	Turn off pumpig system for 40 min then reflow 20 min	Phenobarbtial sodium	Intragastrically administered curcumin (0.825 μmol/kg/day, 0.5 mL per rat)	Intragastrically administered vehicle (0.5 mL olive oil)	15 consecutive days before ischemia	1.AU	1.*p* > 0.05
									2.Initial GSH	2.*p* > 0.05
									3.LDH	3.*p* < 0.05
Liu, H.2017	China	Sprague-Dawley rats male (NM, n = 30/10)	200–250 g	Block LAD for 60 min then reflow for 180 min	Ketamine (90 mg/kg) and xylazine (10 mg/kg)	Curcumin (10/20/30 mg/kg/day)oral	Saline oral	15 days before ischemia	1.IR/LV	1.*p* < 0.01
									2.Caspase-3	2.*p* < 0.01
									3.CAT	3.*p* < 0.01
									4.CK-MB	4.*p* < 0.01
									5.GPx	5.*p* < 0.01
									6.GR	6.*p* < 0.01
									7.LDH	7.*p* < 0.01
									8.MDA	8.*p* < 0.01
									9.SOD	9.*p* < 0.01
Liu, K.2017	China	Sprague-Dawley rats (NM, n = 6/6)	250–300 g	Turn off pumpig system for 30 min then reflow 30 min using Langendorff technique	Chloral hydrate (350 mg/kg)	Curcumin (0.5 mg/kg) myocardial perfusion	Krebs-Henseleit (K-H) solution perfusion	30 min after reperfusion	1.CF	1.*p* < 0.01
									2.CK	2.*p* < 0.01
									3.IR/LV	3.*p* < 0.01
									4.LDH	4.*p* < 0.01
									5.MC	5.*p* < 0.01
									6.STE	6.*p* < 0.01
									7.TNF-α protein	7.*p* < 0.01
Sato, M.2000	US	Sprague-Dawley rats (NM, n = 7/7)	about 300 g	Turn off pumpig system for 30 min then reflow 120 min using Langendorff technique	Pentobarbital sodium (80 mg/kg)	Curcumin (100 μM) myocardial perfusion	Krebs-Henseleit bicarbonate (KHB) solution perfusion	15 min before ischemia	1.IR/LV	1.*p* < 0.05
									2.CF	2.*p* < 0.05
									3.dP/dtmax	3.*p* < 0.05
									4.HR	4.*p* < 0.05
									5.JNK1 protein	5.*p* < 0.05
									6.MAPK	6.*p* < 0.05
									7.p-JNK1	7.*p* < 0.05
Wang, N.P.2012	US	Sprague-Dawley rats (male, n = 8/8)	400–450 g	Block LCA for 45min then reflow for 42 days	Ketamine (90 mg/kg) and zylaxine (10 mg/kg)	Curcumin (150 mg/kg/day) oral	Saline oral	42 days during reperfusion	1. AR/LV	1.*p* < 0.05
									2.Fibrosis/LV	2.*p* < 0.05
									3.LVDd	3.*p*>0.05
									4.LVEF	4.*p* < 0.05
									5.LVFS	5.*p* < 0.05
									6.MDA	6.*p* < 0.05
									7.SV	7.*p* < 0.05
									8.MMP-9	8.*p* < 0.05
Wang N *p*.2014	US	Sprague-Dawley rats (male, n = 8/8)	400–450 g	Block LCA for 30min then reflow for 180min	Ketamine (90 mg/kg) and zylaxine (10 mg/kg)	Curcumin (50 mg/kg/day) oral	Saline oral	5 days before ischemia	1.AR/LV	1.*p* < 0.05
									2.IL-6 protein	2.*p* < 0.05
									3.IR/LV	3.*p* < 0.05
									4.MPO	4.*p* < 0.05
									5.TNF-α protein	5.*p* < 0.05
Wang, R.2021	China	Sprague-Dawley rats (male, n = 6/6)	250–300 g	45 min of ischemia followed by 120 min of reperfusion by using a modi-fied Langendorff technique	Pentobarbital sodium (50 mg/kg)	Curcumin (1 μM) myocardial perfusion	Saline oral	before ischemia	1.IR/AR	1.*p* < 0.01
									2.GSH px	2.*p* < 0.01
									3.LDH	3.*p* < 0.01
									4.MDA	4.*p* < 0.01
									5.SOD	5.*p* < 0.01
Wu,C.H.2011	China	Sprague-Dawley rats (male, 30/10)	200–250 g	Block LAD for 60 min then reflow for 30 min	Pentobarbital sodium (50 mg/kg, 1%)	Transduodenal injection curcumin (10/20/40 mg/kg)	Intravenous injection nothing	30 min earlier before reperfution	1.AST	1.*p* < 0.05
									2.IR/LV	2.*p* < 0.05
									3.LDH	3.*p* < 0.05
									4.LDH1	4.*p* < 0.05
									5.MDA	5.*p* < 0.05
									6.SOD	6.*p* < 0.05
Yang, Y.2013	the U.S.	Sprague-Dawley rats (male, n = 24/8)	220–250 g	Block LAD for 45 min then reflow for 60 min	Pentobarbital sodium (50 mg/kg)	Curcumin (0.25/0.5/1 μM) oral	Saline oral	10 days before ischemia	1.Apoptosis index	1.*p* < 0.01
									2.IR/AR	2.*p* < 0.01
									3.LDH	3.*p* < 0.01
									4.LVDP	4.*p* < 0.01
									5.SIRT1 protein	5.*p* < 0.01
Yao,B.H.2014	China	Sprague-Dawley rats (male, 12/12)	200–250 g	Block LAD for 30 min then reflow for 360 min	Pentobarbital sodium (30 mg/kg, 3%)	Intravenous injection curcumin (20 mg/kg)	Intravenous injection nothing	1 min earlier before reperfution	1.CK	1.*p* < 0.05
									2.LDH	2.*p* < 0.05
									3.Max dp/dt	3.*p* < 0.05
									4.MDA	4.*p* < 0.05
									5.SOD	5.*p* < 0.05
Yeh, C. H. 2005	China (Taiwan)	New Zealand white rabbits (male, n = 20/10)	2.5–3.5 kg	Turn off pumpig system for 1 h then reflow 4 h using Langendorff technique	Pentobarbital sodium (30 mg/kg)	Curcumin (7/70 mmol/kg) injection	Saline injection	2 h before cardiopulmonary bypass	1. IL-6 mRNA	1. *p* < 0.05
									2.MCP-1 mRNA	2.*p* < 0.05
									3.NF-κB protein	3.*p* < 0.05
									4.TNF-α mRNA	4.*p* < 0.05
									5. MMP-2	5. *p* < 0.05
									6. MMP-9	6. *p* < 0.05

AKT, protein kinase B; AR/LV, area in risk/left ventricle; AU, area under the curve; Bcl-2 B-cell lymphoma/Leukemia-2; CF, cardiac flow; CK/CK-MB, creatine kinase; cTnT cardiac troponin T; DMSO, imethyl sulfoxide; dP/dtmax (min), the maximum (minimum) rate of pressure changing the ventricle: GGR GSH/GSSG, ratio; GR, glutathione reductas; GSH, Px glutathione peroxidase; HR, heart rate; IR/AR, infarction region/area in risk; IR/LV, infarction region/left ventricle; ISR, ischemic region; LAC, the left coronaryarter; LDH, lactate dehydrogenase; LVDd, left ventricular end diastolic diameter; LVDP, left ventricular peak developing pressure; LVEF, left ventricular ejection fraction; LVFS, left ventricular fraction shortening; LVSd, left ventricular end systolic diameter; MA, myocardial apoptosis; MAP, mean arterial pressure; MAPK, p38 mitogen-activated protein kinase; MC, myocardial contractility; MDA, malondialdehyde; MIS, myocardial infarction size; MPO, myeloperoxidase; NF-κB, nuclear factor kappa B; NM, no mention; NOAC, the number of apoptosis cells; OC, oxygen consumption; p-JNK1 phosphorylated c-Jun NH2-terminal kinase; PLVN, percentage left ventricle necrosis; SIRT1 sirtuin1; SMA, superior mesenteric artery; SOD, superoxide dismutase; STE ST-segment elevation; SV, stroke volume; VF, ventricular fibrillation; VPB, ventricular premature beats; VT, ventricular tachycardia.

#### 3.2.2 Human studies

All human studies (Wongcharoen et al., 2011; Wongcharoen et al., 2012; Aslanabadi et al., 2019; Phrommintikul et al., 2019) are English literature studies, mainly distributed in Iran and Thailand. All 435 patients included in the study were ischemic heart disease patients who needed PCI or CABG treatment, and were randomly divided into curcumin treatment group and conventional treatment group. The clinical characteristics are summarized in Table 2.

**TABLE 2:** Basic characteristics of the four included human studies.

Study ID	Country	Male/female	Number of experimental group	Number of control group	Treatment group (method to curcumin)	Control group	Duration	Time of following-up	Outcomes	*p*-Value
Arintaya Phrommintikul. 2019	Thailand	69/31	50	50	Curcumin treatment: curcumin nanomicelle 4 g per day P.O for 1 day before PCI and 1day post PCI Conventional treatment: Not mention	Conventional treatment: Not mention	2 days	48 h after PCI	1.hs-TnT after PCI	1.*p* = 0.912
									2.hs-CRP after PCI	2.*p* = 0.873
Naser Aslanabadi. 2019	Iran	66/44	55	55	Curcumin treatment: curcumin nanomicelle 480 mg P.O at 1–2 h before PCI Conventional treatment: aspirin 325 mg and clopidogrel 300 mg orally plus weight-adjusted intravenous heparin with a target activated clotting time 250–350 s	Pretreatment: aspirin 325 mg and clopidogrel 300 mg orally plus weight-adjusted intravenous heparin with a target activated clotting time 250–350 s	once	8 and 24 h after PCI	1.CK-MB 8 h after PCI	1.*p* = 0.24
									2.CK-MB 24 h after PCI	2.*p* = 0.37
									3.cTnI 8 h after PCI	3.*p* = 1.0
									4.cTnI 24 h after PCI	4.*p* = 0.35
Wanwarang Wongcharoen. 2012	Thailand	69/52	61	60	Curcumin treatment: curcumin nanomicelle 4 g per day P.O for 3 days before CABG and 5 days post CABG Conventional treatment: Not mention	Conventional treatment: Not mention	8 days	30 days after CABG	1.CRP 3 days after CABG	1.*p* = 0.031
									2.CRP 5 days after CABG	2.*p* > 0.05
									3.MDA 5 days after CABG	3.*p* < 0.001
									4. Incidence of in-hospital MI	4.*p* = 0.028
									5. Incidence of left ventricular dysfunction	5.*p* = 0.021
									6.NT-proBNP	6.0.015
W. Wongcharoen. 2011	Thailand	Not mention	52	52	Curcumin treatment: curcumin nanomicelle 4 g per day P.O for 3 days before CABG and 7 days post CABG Conventional treatment: Not mention	Conventional treatment: Not mention	10 days	30 days after CABG	1. Incidence of in-hospital MI	1.*p* < 0.05
									2. Incidence of MACE	2.*p* < 0.05
									3.CK-MB after CABG	3.*p* < 0.05

CABG, coronary artery bypass grafting; CK-MB, creatine kinase; MB, form; cTnI, cardiac troponin; hs-CRP, high-sensitive cardiac troponin T; hs-TnT, high-sensitive cardiac troponin T; MACE, major adverse cardiovascular events; MDA, malonaldehyde; MI, myocardium infarction; P.O, peros; PCI, percutaneous coronary intervention.

### 3.3 Study quality

#### 3.3.1 Animal studies

We evaluated the quality of all included studies. The score of study quality ranged from two to six with a total of ten points. One study (Hardy et al., 2015) got two points, eight studies (Cheng et al., 2005; Yeh et al., 2005; Ali et al., 2009; Yang et al., 2013a; Leong et al., 2013; Chen et al., 2016; Gu et al., 2016; Deng et al., 2018) got three points, four studies (Sato et al., 2000; Duan et al., 2012; Liu et al., 2017a; Wang et al., 2018) got four points, five studies (Kim et al., 2008; Gonzalez-Salazar et al., 2011; Broskova et al., 2013; Liu et al., 2017b; Jo et al., 2020) got five points, and the remaining 6 (Wu, 2011; Jeong et al., 2012; Kim et al., 2012; Wang et al., 2012; Wang et al., 2014; Yao and Jiang, 2014) got six points. All studies reported random allocation of animals, but only 1 (Gu et al., 2016) of them reported the process of random sequence generation. Five studies (Yeh et al., 2005; Yang et al., 2013a; Leong et al., 2013; Hardy et al., 2015; Gu et al., 2016) did not fully report on how to balance baseline characteristics. Twelve studies (Sato et al., 2000; Cheng et al., 2005; Yeh et al., 2005; Ali et al., 2009; Duan et al., 2012; Yang et al., 2013a; Leong et al., 2013; Hardy et al., 2015; Chen et al., 2016; Gu et al., 2016; Liu et al., 2017a; Deng et al., 2018) did not report allocation concealment. Eight studied (Sato et al., 2000; Wu, 2011; Kim et al., 2012; Wang et al., 2012; Broskova et al., 2013; Wang et al., 2014; Yao and Jiang, 2014; Liu et al., 2017a) reported randomization of animal placement. No studies reported the implementation of blind methods for caregivers or experimental researchers. Thirteen studies (Yeh et al., 2005; Kim et al., 2008; Gonzalez-Salazar et al., 2011; Wu, 2011; Jeong et al., 2012; Kim et al., 2012; Wang et al., 2012; Yang et al., 2013a; Leong et al., 2013; Wang et al., 2014; Yao and Jiang, 2014; Liu et al., 2017b; Jo et al., 2020) used a random outcome assessment. Only one study (Duan et al., 2012) mentioned the blinding outcome assessor, while the others did not blind the assessors. No study reported incomplete data. Only one study (Jeong et al., 2012) had the study protocol, which showed no selective outcome reporting occurred, while the others remained unclear. All studies had no other sources of bias. The overall methodological quality of the included animal studies is shown in Table 3.

**TABLE 3:** Methodological quality of included animal studies (SYRCLE tool).

Study ID	A	B	C	D	E	F	G	H	I	J	Scores
Ali, M. S. 2009	0	1	0	0	0	0	0	1	0	1	3
Brosková, Z. 2013	0	1	1	1	0	0	0	1	0	1	5
Chen, X. L. 2016	0	1	0	0	0	0	0	1	0	1	3
Cheng, H. 2005	0	1	0	0	0	0	0	1	0	1	3
Deng, Y. 2018	0	1	0	0	0	0	0	1	0	1	3
Duan, W. 2012	0	1	0	0	0	0	1	1	0	1	4
González-Salazar, A. 2011	0	1	1	0	0	1	0	1	0	1	5
Gu, H. P. 2016	1	0	0	0	0	0	0	1	0	1	3
Hardy, N. 2015	0	0	0	0	0	0	0	1	0	1	2
Jeong, C. W. 2012	0	1	1	0	0	1	0	1	1	1	6
Jo, W. 2020	0	1	1	0	0	1	0	1	0	1	5
Kim, Y. S. 2008	0	1	1	0	0	1	0	1	0	1	5
Kim, Y. S. 2012	0	1	1	1	0	1	0	1	0	1	6
Leong, P. K. 2013	0	0	0	0	0	1	0	1	0	1	3
Liu, H. 2017	0	1	1	0	0	1	0	1	0	1	5
Liu, K. 2017	0	1	0	1	0	0	0	1	0	1	4
Sato, M. 2000	0	1	0	1	0	0	0	1	0	1	4
Wang N. P. 2014	0	1	1	1	0	1	0	1	0	1	6
Wang, N. P. 2012	0	1	1	1	0	1	0	1	0	1	6
Wang, R. 2021	0	1	1	0	0	0	0	1	0	1	4
Wu, C. H. 2011	0	1	1	1	0	1	0	1	0	1	6
Yang, Y. 2013	0	0	0	0	0	1	0	1	0	1	3
Yao, B. H. 2014	0	1	1	1	0	1	0	1	0	1	6
Yeh, C. H. 2005	0	0	0	0	0	1	0	1	0	1	3

A, sequence generation; B, baseline characteristics; C, allocation concealment; D, random housing; E, blinding investigators; F, random outcome assessment; G, blinding outcome assessor; H, incomplete outcome data; I, selective outcome reporting; J, other sources of bias.

#### 3.3.2 Human studies

RoB-2 tool was used to evaluate the methodological quality (deviation risk) of the four included human studies. Two of the studies were judged as low risk, while the other two were judged as some concerns. The details are shown in Table 4.

**TABLE 4:** Methodological quality of included human studies (RoB-2 tool).

Study ID	Randomization process	Assignment to intervention	Adhering to intervention	Missing outcome data	Measurement of outcome	Selection of the reported result	RoB-2 overall score
Arintaya Phrommintikul. 2019	Low risk	Low risk	Low risk	Low risk	Low risk	Low risk	Low risk
Naser Aslanabadi. 2019	Low risk	Some concerns	Low risk	Some concerns	Low risk	Some concerns	Some concerns
Wanwarang Wongcharoen. 2012	Low risk	Low risk	Low risk	Low risk	Low risk	Low risk	Low risk
Wanwarang Wongcharoen. 2011	Some concerns	Low risk	Low risk	Low risk	Low risk	Some concerns	Some concerns

### 3.4 Data analysis of animal studies

#### 3.4.1 Myocardial infarction size

**FIGURE 3:**
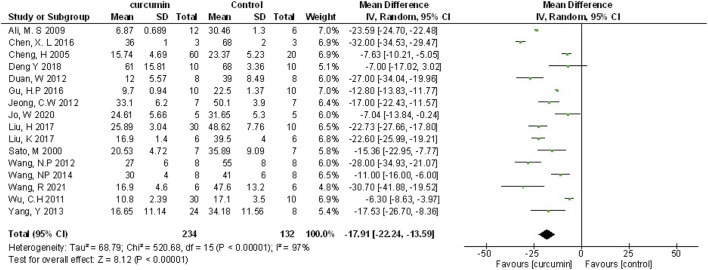
The meta-analysis results of the effect of curcumin on MI size.

Myocardial infarction (MI) size was calculated by infarct area/left ventricular area. A meta-analysis of 16 animal studies demonstrated that curcumin significantly decreased MI size compared with the control group (n = 246, WMD = −17.91%, 95% CI (−22.24%, −13.59%), *p* < 0.00001, I^2^ = 97%) (Figure 3). Because of the obvious heterogeneity in the included animal studies, we performed a sensitivity analysis. After removing the animal studies one by one, we failed to determine the source of heterogeneity, but the sensitivity analysis indicated that the outcome was steady.

##### 3.4.1.1 Dose-response meta-analysis of curcumin

**FIGURE 4:**
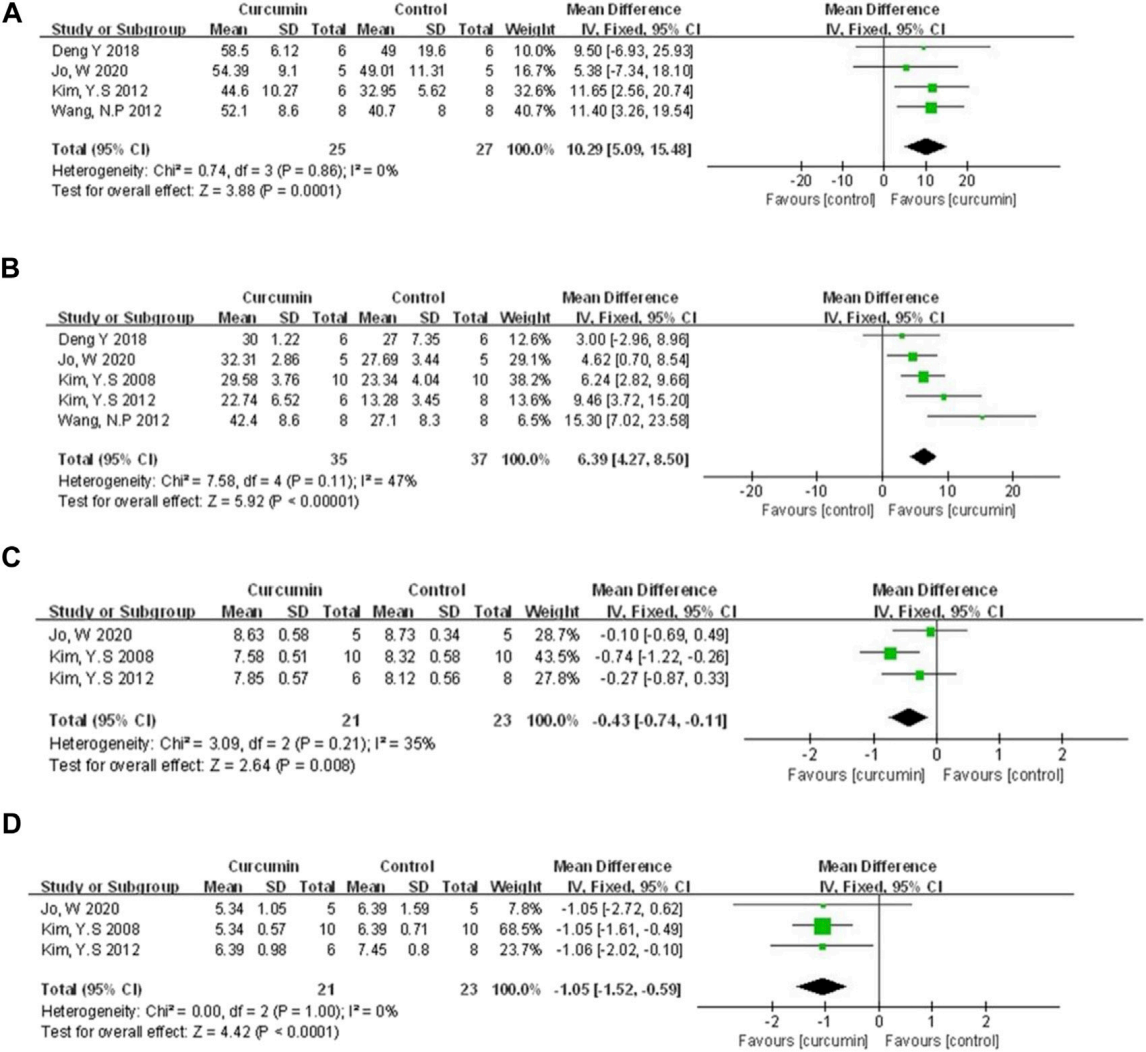
**(A)** The meta-analysis results of the effect of curcumin on LVEF **(B)** The meta-analysis results of the effect of curcumin on LVFS. **(C)** The meta-analysis results of the effect of curcumin on LVDd. **(D)** The meta-analysis results of the effect of curcumin on LVSd.

According to previous studies, different doses of curcumin could affect the efficacy (Yang et al., 2013a; Liu et al., 2017b), which might be one of the main reasons for the high outcome heterogeneity. Therefore, we performed a dose-response meta-analysis of animal studies (Jeong et al., 2012; Wang et al., 2012; Yang et al., 2013a; Wang et al., 2014; Chen et al., 2016; Liu et al., 2017b; Jo et al., 2020) in which curcumin was administered orally. Firstly, RR was recalculated and defined as the ratio of means (means_experimental_/means_control_). Then, scatter plot was drawn for curcumin dose and RR (Supplementary Figure S1). Through image analysis, the point distribution showed a non-linear relationship. We hypothesized that a non-linear relationship might be applicable to the relationship between dose and the protective effect of curcumin. Non-linear regression was conducted according to the quadratic regression method of meta-analysis (Orsini et al., 2012). with the increase of curcumin dosage, the protective effect of curcumin on myocardial I/R was firstly enhanced, then weakened and then strengthened (Figure 4). In our model, 200 mg/kg BW per day was the optimal dose of curcumin (when dosage ranges from 10 to 200 mg/kg BW per day) and had the best predictive protective effect, and the prediction relationship can be expressed as LnRR = −1*10^−6^ x^3^ + 0.0003 x^2^ − 0.0205 x − 0.2386, *R* ^2^ = 0.638.

#### 3.4.2 Cardiac function

Left ventricular ejection fraction (LVEF), left ventricular fraction shortening (LVFS), left ventricular end-diastolic dimension (LVDD), left ventricular end-systolic dimension (LVSD) were examined to demonstrate improvement in cardiac function induced by curcumin. A total of four animal studies (Kim et al., 2012; Wang et al., 2012; Deng et al., 2018; Jo et al., 2020) reported the protective effects of curcumin on LVEF compared to the control group (n = 52, WMD = 10.29%, 95% CI (5.09%, −15.48%), *p* = 0.0001, I2 = 0%) (Figure 4A). Results from five animal studies (Kim et al., 2008; Kim et al., 2012; Wang et al., 2012; Deng et al., 2018; Jo et al., 2020) showed curcumin could increase LVFS compared to the control group (n = 72, WMD = 6.39%, 95% CI (4.27%, 8.50%), *p* < 0.00001, I2 = 47%) (Figure 4B). Results from three animal studies (Kim et al., 2008; Kim et al., 2012; Jo et al., 2020) showed curcumin could decrease LVDD compared to the control group (n = 44, WMD = −0.43, 95% CI (−0.74, −0.11), *p* = 0.008, I2 = 35%) (Figure 4C). Results from three animal studies (Kim et al., 2008; Kim et al., 2012; Jo et al., 2020) showed curcumin could decrease LVSD compared to the control group (n = 44, WMD = −1.05, 95% CI (−1.52, −0.59), *p* < 0.0001, I2 = 0%) (Figure 4D).

#### 3.4.3 Myocardial injury marker

**FIGURE 5:**
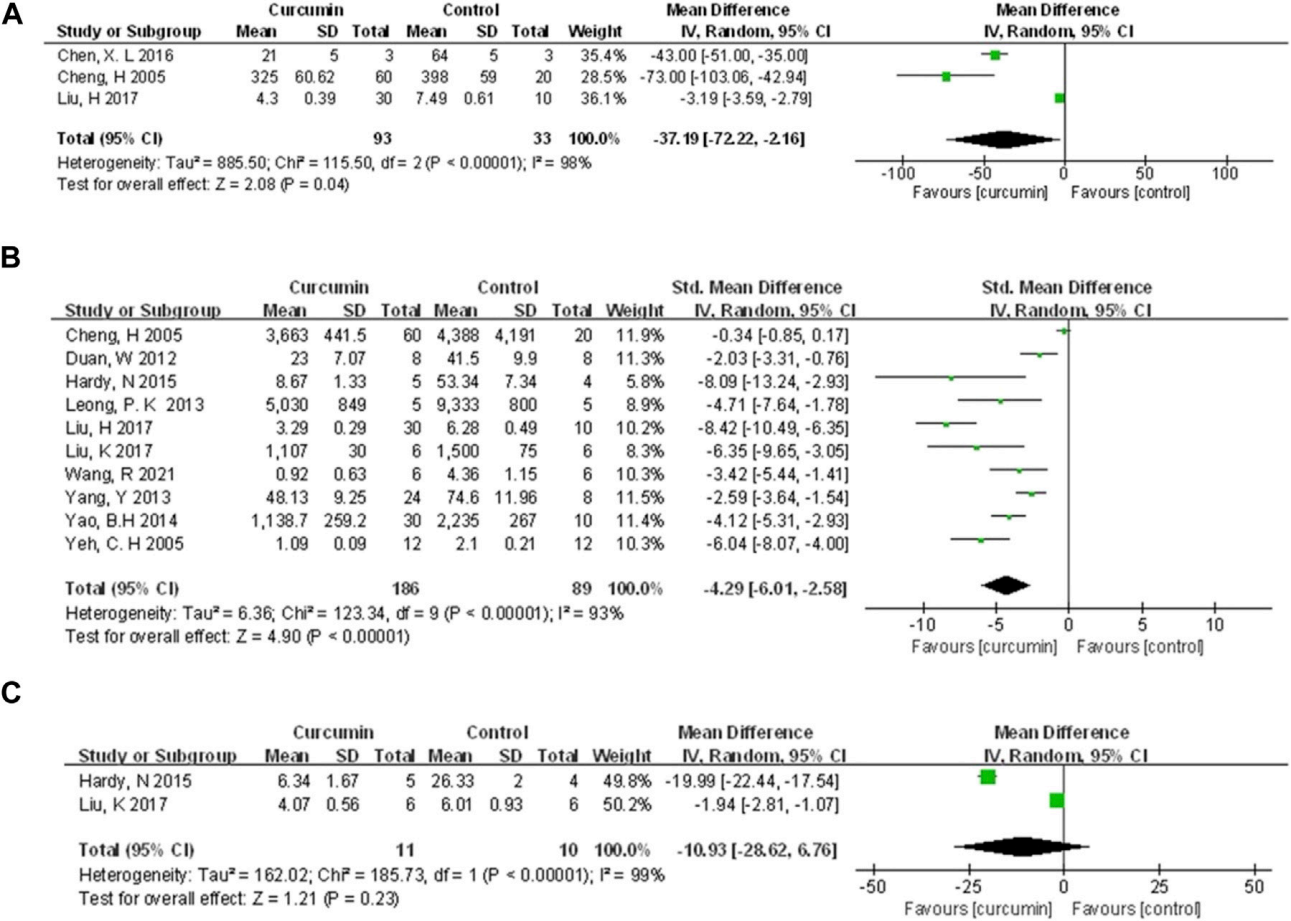
The meta-analysis results of the effect of curcumin on **(A)** CK-MB **(B)** LDH, and **(C)** CK.

Compared with the control group, meta-analysis of three animal studies (Cheng et al., 2005; Chen et al., 2016; Liu et al., 2017b) showed curcumin can reduce CK-MB (n = 126, WMD = −37.19, 95% CI (−72.22, −2.16), *p* = 0.04, I2 = 98%) (Figure 5A). A meta-analysis of 10 animal studies (Cheng et al., 2005; Yeh et al., 2005; Duan et al., 2012; Yang et al., 2013a; Leong et al., 2013; Yao and Jiang, 2014; Hardy et al., 2015; Liu et al., 2017a; Liu et al., 2017b; Wang et al., 2018) showed curcumin can reduce LDH (n = 275, SMD = −4.29, 95% CI (−6.01, −2.58), *p* < 0.00001, I2 = 93%) (Figure 5B). Through removing the animal studies one by one, the sensitivity analysis indicated that the outcome was steady. The pooled results of two animal studies (Hardy et al., 2015; Liu et al., 2017a) on CK did not show difference between curcumin and control group (n = 21, WMD = −10.93, 95% CI (−28.62, 6.76), *p* = 0.23, I2 = 99%) (Figure 5C). Moreover, compared with the control group, one study (Gu et al., 2016) showed curcumin can decrease cTnT (*p* < 0.05), and another one (Kim et al., 2008) showed curcumin can decrease MPO (*p* < 0.05).

#### 3.4.4 Cardioprotective mechanisms

**FIGURE 6:**
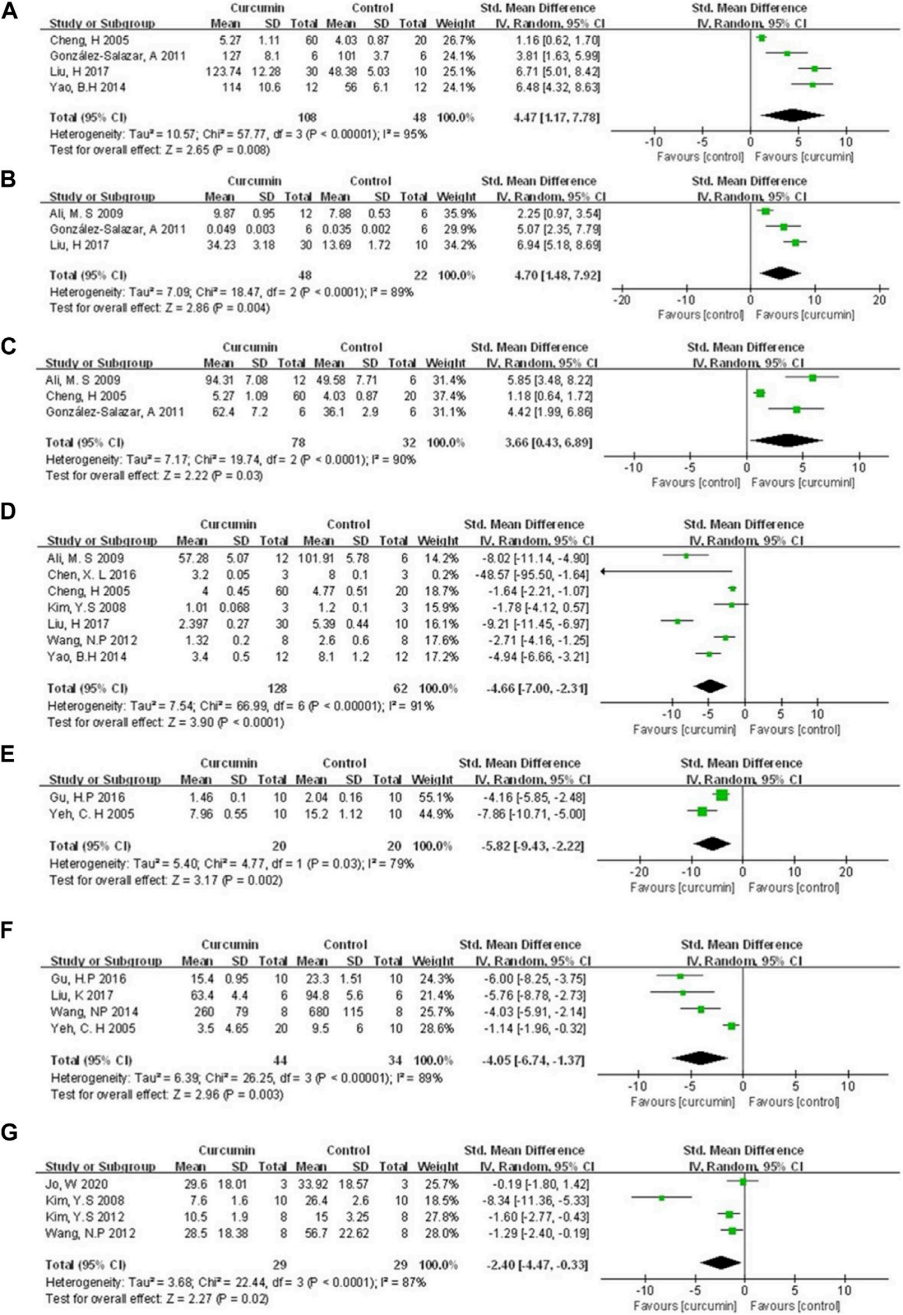
The meta-analysis results of the effect of curcumin on **(A)** SOD **(B)** CAT **(C)** GSH **(D)** MDA **(E)** NF-κB protein **(F)** TNF-α protein, and **(G)** Fibrosis/LV%.

The cardioprotective protective mechanisms involved in our study included cardiomyocyte inflammation, oxidation, apoptosis, myocardial fibrosis, and et al. A meta-analysis of four animal studies (Cheng et al., 2005; Gonzalez-Salazar et al., 2011; Yao and Jiang, 2014; Liu et al., 2017b) showed that curcumin had a significant effect on increasing SOD expression compared with the control group (n = 156, SMD = 4.47, 95% CI (1.17, 7.78), *p* = 0.008, I2 = 95%) (Figure 6A). A meta-analysis of three animal studies (Ali et al., 2009; Gonzalez-Salazar et al., 2011; Liu et al., 2017b) showed that curcumin had a significant effect on increasing CAT expression compared with the control group (n = 70, SMD = 4.70, 95% CI (1.48, 7.92), *p* = 0.004, I2 = 89%) (Figure 6B). Three animal studies (Cheng et al., 2005; Ali et al., 2009; Gonzalez-Salazar et al., 2011) showed that curcumin could increase GSH expression compared with the control group (n = 110, SMD = 3.66, 95% CI (0.43, 6.89), *p* = 0.03, I2 = 90%) (Figure 6C). A meta-analysis of seven animal studies (Cheng et al., 2005; Kim et al., 2008; Ali et al., 2009; Wang et al., 2012; Yao and Jiang, 2014; Chen et al., 2016; Liu et al., 2017b) showed that curcumin has a significant effect on reducing MDA expression compared with the control group (n = 190, SMD = −4.66, 95% CI (−7.00, −2.31), *p* < 0.0001, I2 = 91%) (Figure 6D). A meta-analysis of two animal studies (Yeh et al., 2005; Gu et al., 2016) indicated that curcumin can suppress the expression of NF-κB protein compared with the control group (n = 40, SMD = −5.82, 95% CI (−9.43, −2.22), *p* = 0.002, I2 = 79%) (Figure 6E). Results from four animal studies (Yeh et al., 2005; Wang et al., 2014; Gu et al., 2016; Liu et al., 2017a) showed curcumin could decrease TNF-α protein compared to the control group (n = 78, SMD = −4.05, 95% CI (−6.74, −1.37), *p* = 0.003, I2 = 89%) (Figure 6F). Results from four animal studies (Kim et al., 2008; Kim et al., 2012; Wang et al., 2012; Jo et al., 2020) showed curcumin could decrease the area of fibrosis compared to the control group (n = 58, SMD = −2.40, 95% CI (−4.47, −0.33), *p* = 0.02, I2 = 87%) (Figure 6G).

Because of the small number of animal studies (<3 studies), other mechanism indicators reflecting the cardioprotective function of curcumin only are described as follows. Unless otherwise specified, these reports indicated positive effects of the curcumin group on the mechanism of anti-myocardial I/R injury (*p*-value indicates comparison with the control group). These indicators included Bcl-2 (Duan et al., 2012; Deng et al., 2018) (*p* < 0.05), Caspase-3 (Chen et al., 2016; Liu et al., 2017b) (p < 0.05 or *p* < 0.01), MMP-9 (Yeh et al., 2005; Wang et al., 2012) (*p* < 0.05), MPO (Kim et al., 2008; Wang et al., 2014) (*p* < 0.05), IL-6 mRNA (Yeh et al., 2005; Wang et al., 2014) (*p* < 0.05). The included animal studies also reported the regulation of curcumin on other proteins. However, because the frequency of occurrence was <2, they would not be described in detail (All reported indicators can be found in Table 1).

#### 3.4.5 Subgroup analysis and meta-regression

**FIGURE 7:**
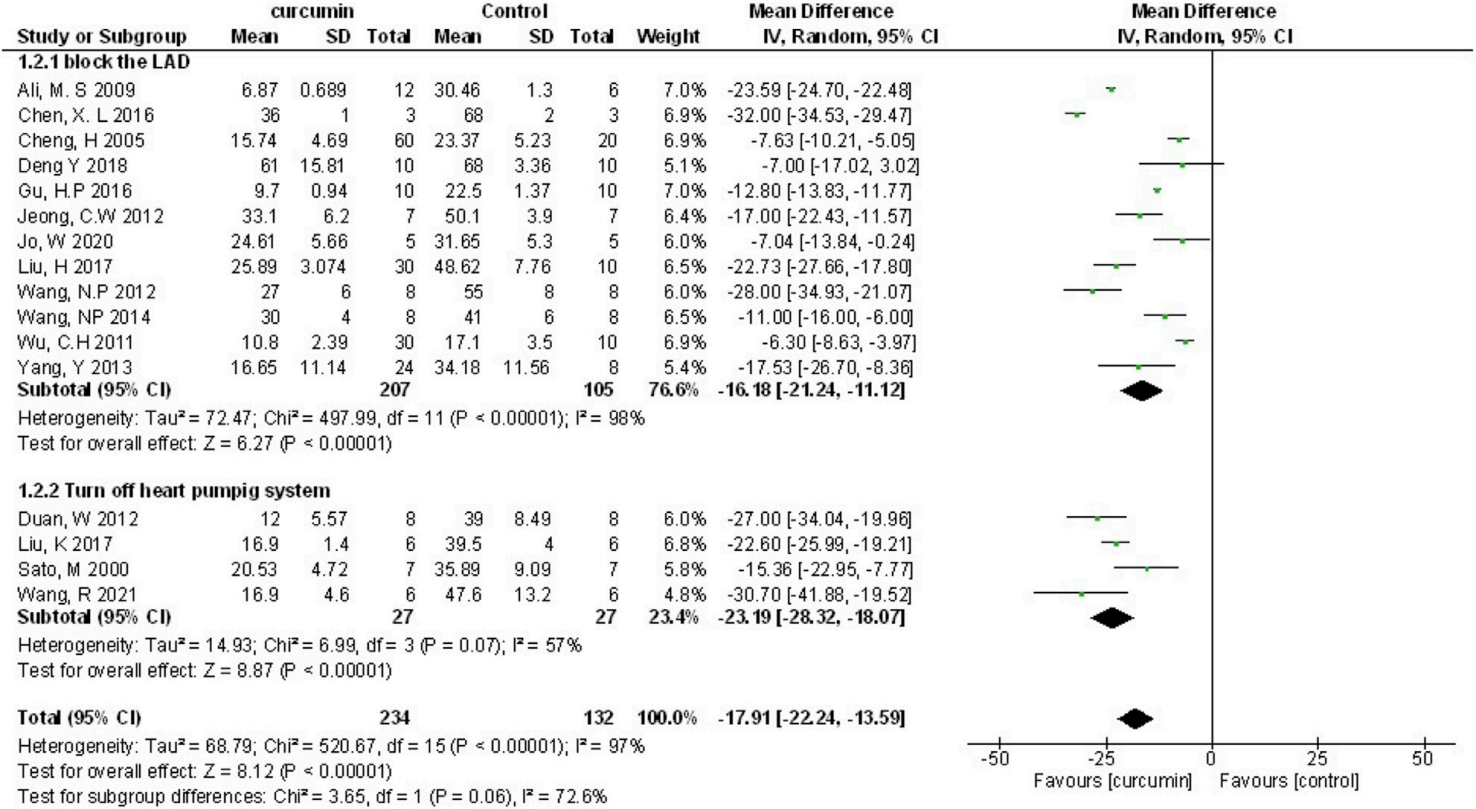
The subgroup analysis (method of model establishment) of curcumin on MI size.

**FIGURE 8:**
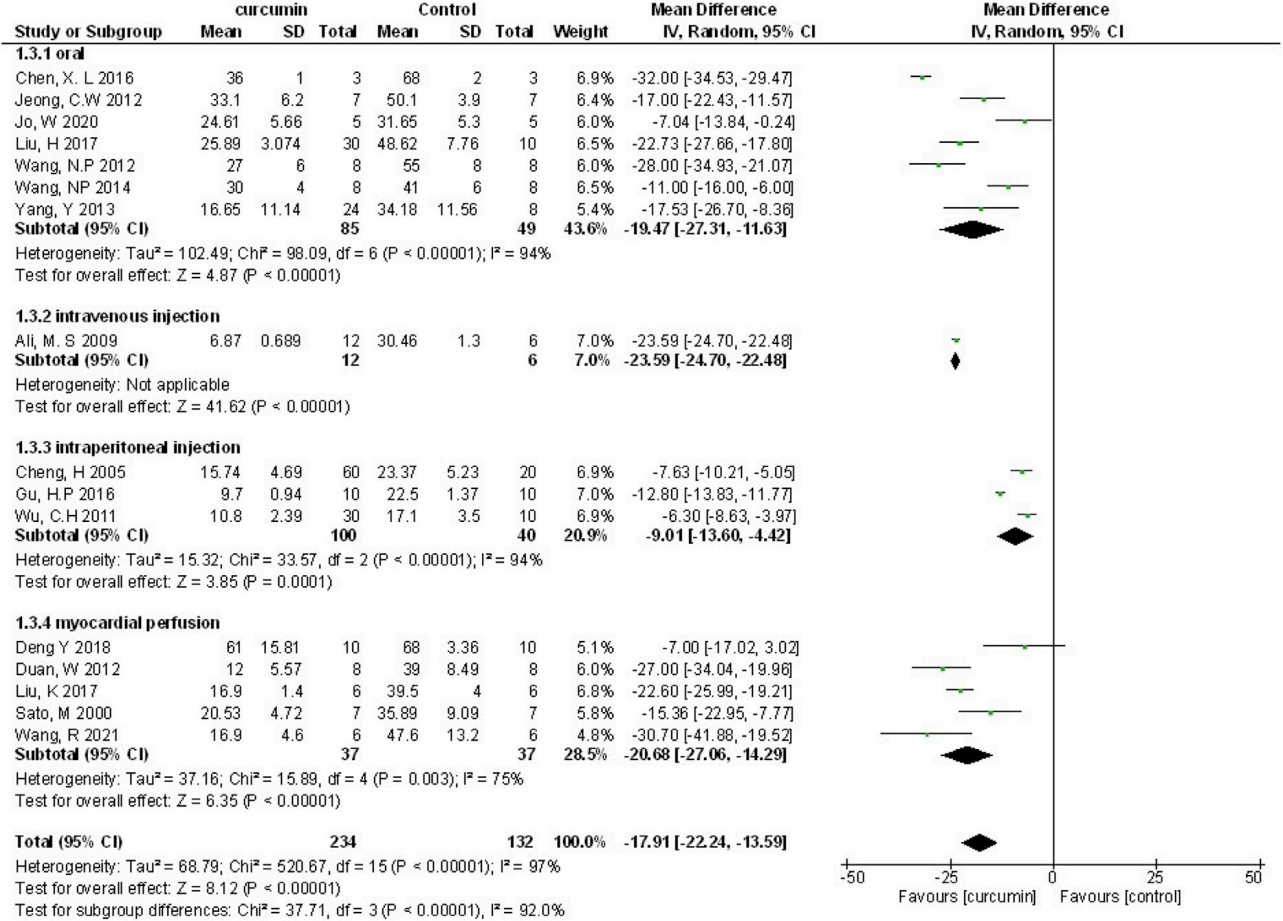
The subgroup analysis (administration method) of curcumin on MI size.

**FIGURE 9:**
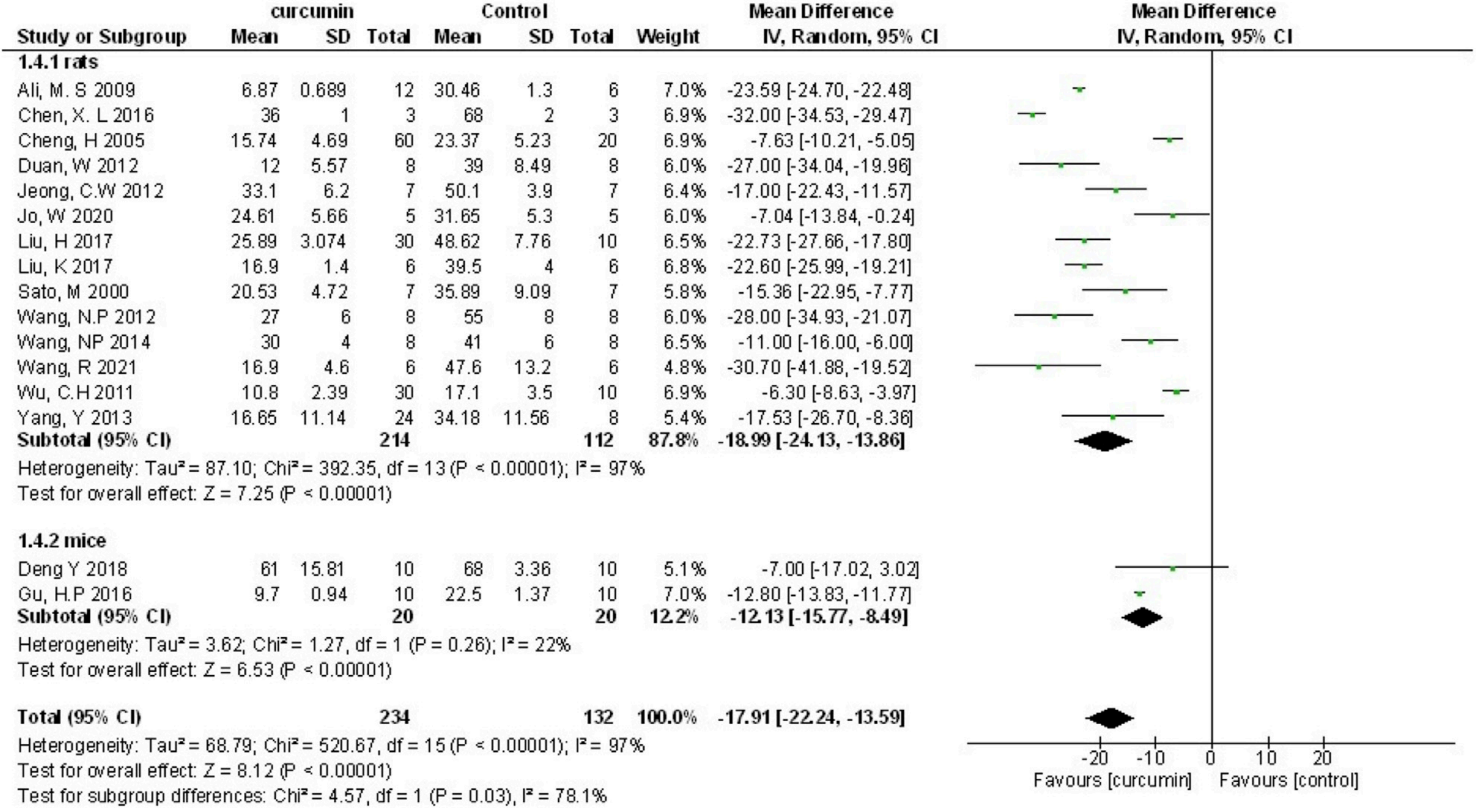
The subgroup analysis (genus of experimental animals) of curcumin on MI size.

**FIGURE 10:**
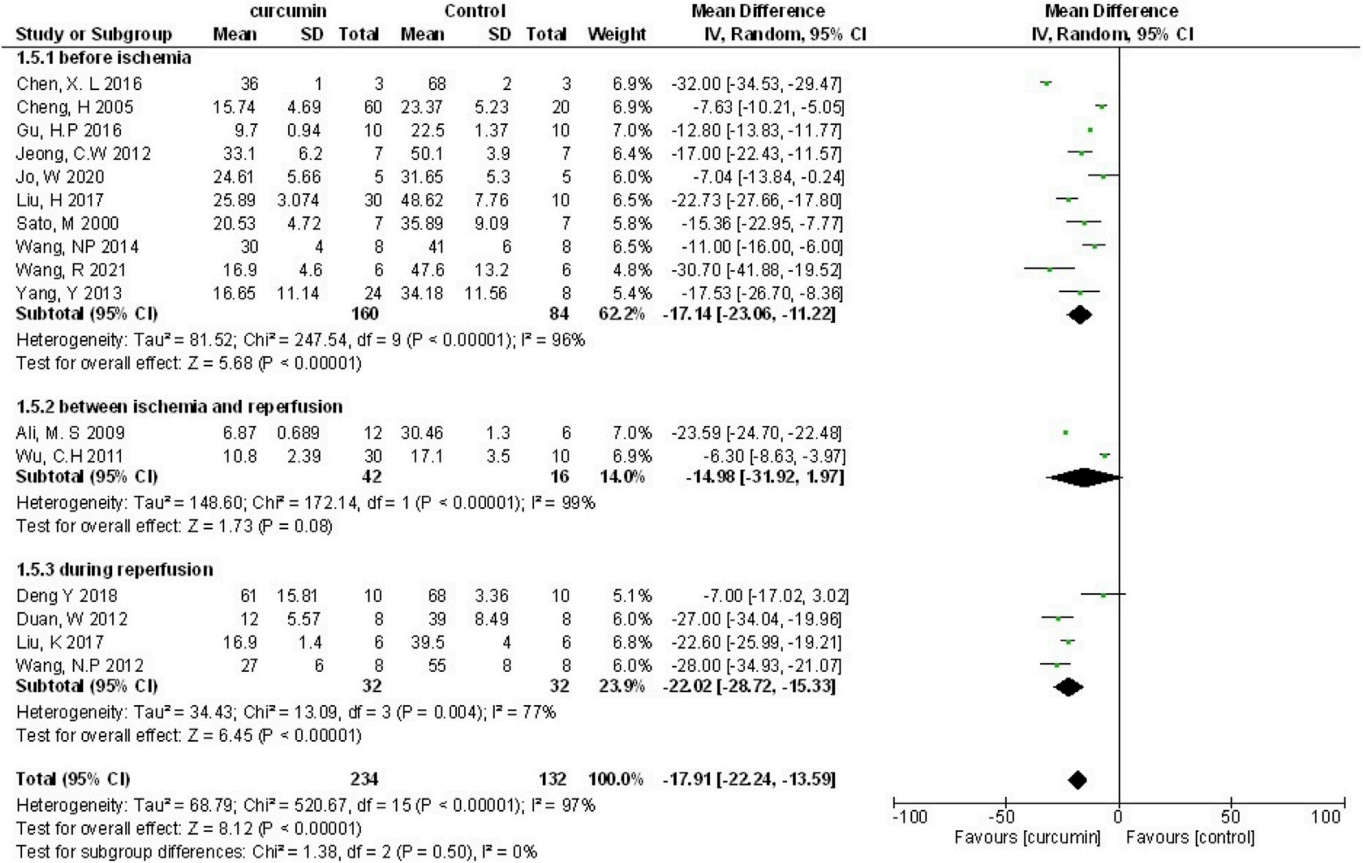
The subgroup analysis (intervention moment) of curcumin on MI size.

We performed the subgroup analysis on primary outcomes according to preset subgroups. The LVEF, LVFS, LVDD, LVSD in the primary outcome indicators were not included in the subgroup analysis because of not enough animal studies, so we only carried out the subgroup analysis on MI size. Analysis of the three preset subgroups (method of model establishment, administration method, and genus of experimental animals) indicated outcomes that were consistent with the overall rusults (*p* < 0.05) (Figures 7–9). Some outcomes of the subgroup analysis by intervention moment (before ischemia/between ischemia and reperfusion/during reperfusion) were inconsistent with the overall results (Figure 10). The subgroup analyzed for before ischemia group and during reperfusion group were consistent with the overall results (*p* < 0.05), while the outcome of between ischemia and reperfusion group was inconsistent with the overall results (*p* > 0.05). Moreover, I2 did not decrease, suggesting that the preset subgroups did not explain the source of heterogeneity of MI size.

To further explore the source of heterogeneity, we conducted a meta-regression analysis. Eight characteristics were selected for meta-regression, including: 1) Region (US, China, India, or South Korea), 2) year of publication (2000–2016, 2017–2021), 3) whether the original study set the dose subgroup, 4) In vivo or ex-vivo experiments, 5) myocardial ischemia duration (30min, 45min or 60min), 6) times of administration (single-dose or multiple-dose), 7) No. of sample size (<20 or ≥20), 8) publication language (English or Chinese). However, meta-regression analysis (Table 5) did not reveal a significant impact of the covariates above, which indicated none of the above characteristics was the source of heterogeneity between animal studies.

**TABLE 5:** Meta-regression analysis of potential sources of animal studies heterogeneity.

Heterogeneity factor	Coefficient	SE	t	*p-*value	95% CI (lower limit, upper limit)
region	4.255238	2.975009	1.43	0.196	−2.77954, 11.29002
year of publication	3.702559	4.491461	0.82	0.437	−6.91806, 14.32318
whether thestudy set the dose subgroup	4.001104	5.08711	0.79	0.457	−8.027999, 16.03021
*in vivo* or *in vitro* experiments	−0.779716	5.795373	−0.13	0.897	−14.4836, 12.92416
myocardial ischemia duration	1.391345	2.765627	0.50	0.630	−5.148324, 7.931014
times of administration	0.21975	5.388646	0.04	0.969	−12.52237, 12.961873
animal sample size	2.58827	5.942484	0.44	0.676	−11.46347, 16.64001
publication language	3.311636	7.378133	0.45	0.667	−14.13488, 20.75815
Cutoff value	−35.80981	21.23304	−1.69	0.136	−86.01798, 14.39835

SE, standard error; CI, confidence interval.

#### 3.4.5 Publication bias

**FIGURE 11:**
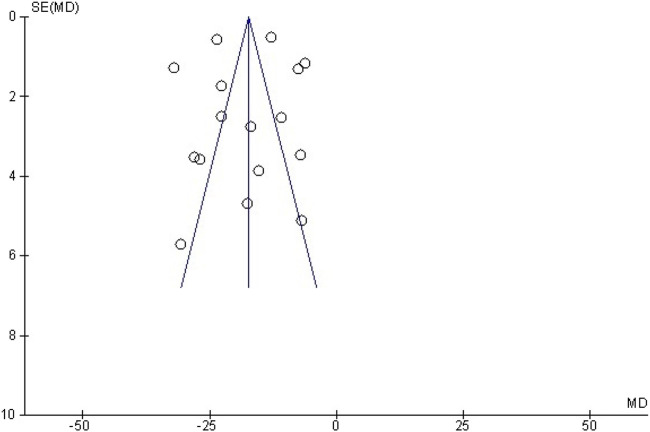
Funnel plot detailing publication bias in animal studies reporting the effect of curcumin on MI size.

A funnel plot was adopted to assess the publication bias regarding myocardial infarction size (Figure 11). Furthermore, we used Egger’s regression test to check out the asymmetry of the graph, and the results indicated no publication bias in this study (*p* = 0.92 > 0.05).

### 3.5 Systematic analysis of human studies

#### 3.5.1 Methods and characteristics of curcumin treatment

Oral curcumin nanomicelle was used in four human studies. Two studies (Aslanabadi et al., 2019; Phrommintikul et al., 2019) used a shorter course of treatment (1–2 days), with a total amount of 0.48g–8 g of curcumin. The other two studies (Wongcharoen et al., 2011; Wongcharoen et al., 2012) used a longer course of treatment (8–10 days), with a total amount of 32g–40 g of curcumin. All the curcumin treatment groups in the study were combined with conventional treatment, and only one study described the conventional treatment protocol in detail.

#### 3.5.2 Biomarkers of myocardial injury

cTnI, cTnT and CK-MB have been used clinically for a long time to evaluate the myocardial injury in patients. Three studies (Wongcharoen et al., 2011; Aslanabadi et al., 2019; Phrommintikul et al., 2019) detected serum biomarkers of myocardial injury after revascularization, of which two (Aslanabadi et al., 2019; Phrommintikul et al., 2019) showed no statistically significant difference between curcumin treatment group and control group, hs TnT (201 ± 547 vs 187 ± 703.9 ng/L, *p* = 0.912), cTnI (180 ± 190 vs 220 ± 190 ng/L, *p* = 0.35), CK-MB (21.8 ± 5.4 vs 23.1 ± 8.5 U/L, *p* = 0.37). One study (Wongcharoen et al., 2011) showed that curcumin had a therapeutic effect in reducing CK-MB after revascularization (43 ± 18 vs 58 ± 44 ng/mL, *p* = 0.02).

#### 3.5.3 Biomarkers of inflammation and oxidative stress injury

Serum CRP and MDA were used to evaluate the degree of inflammation and oxidative stress injury. The results of study (Phrommintikul et al., 2019) showed that there was no statistically significant difference in CRP reduction between the curcumin group and the control group (7.2 ± 18.8 vs 6.6 ± 17.5 mg/dL, *p* = 0.87), but the results of study (Wongcharoen et al., 2012) showed that compared with the control group, the curcumin group could reduce CRP (161.8 ± 54.1 vs 128.6 ± 60.5 mg/dL, *p* = 0.031) and MDA (0.8 ± 1.4 vs 5.7 ± 1.5 mmol/mL, *p* = 0.001).

#### 3.5.4 Cardiac function, incidence of in-hospital MI and MACE within 30 days after revascularization

For patients with ischemic heart disease, in-hospital MI and MACE after revascularization are the most important clinical outcome, and cardiac function is the most important factor affecting the above outcome. The study (Wongcharoen et al., 2012) showed that compared with the control group, the curcumin group could reduce the incidence of left ventricular dysfunction, LVEF<40% (3.3% vs 25.9% *p* = 0.021), and NT-proBNP (1822.1 ± 2,102.9 vs 2,542.2 ± 2,631.2 pg/mL, *p* = 0.015). The results of two studies (Wongcharoen et al., 2011; Wongcharoen et al., 2012) showed that the incidence of in-hospital MI in the curcumin group was significantly lower than that in the control group. The study (Wongcharoen et al., 2011) showed that compared with the control group, the incidence of MACE within 30 days after revascularization in the curcumin group was lower (13.5% vs. 34.6%, *p* = 0.02).

## 4 Discussion

### 4.1 Contradiction between human studies and animal studies

This is the first systematic review and meta-analysis consisting of preclinical and clinical evidence to investigate the cardioprotective effects of curcumin on myocardial I/R injury. Twenty-four studies with 503 animals and four human studies with 435 patients were included, and the overall methodological quality of the included studies was moderate. The results of animal studies meta-analysis suggested that curcumin might diminish myocardial infarction size, improve heart function, suppress biomarkers concentration for myocardial infarction, as well as ameliorate cardiomyocyte inflammation, oxidation, apoptosis, and myocardial fibrosis in animal studies.

Meta-analysis of animal studies shown the clinically potential of curcumin in the treatment of myocardial I/R injury. Interestingly, some of results of human studies showed different opinions. The results of two studies (Aslanabadi et al., 2019; Phrommintikul et al., 2019) showed that there was no statistically significance between curcumin group and control group in serum myocardial injury biomarkers (hscTnT, cTnI, CK-MB) after PCI or CABG. However, we found that in N. Aslanabadi et al.’s study (Aslanabadi et al., 2019), the incidence of CK-MB rising above the normal value during PCI in curcumin group was 50% less than that in control group. In addition, the decrease of serum CK-MB in patients in curcumin group 24 h after PCI were more notable than that in the control group (2.6 ± 8.7 vs 0.29 ± 8.8 U/L, *p* = 0.75). Although *p* > 0.05, both indicators showed descending trend. Besides, it should be noted that the protocol of curcumin treatment in these two studies (Aslanabadi et al., 2019; Phrommintikul et al., 2019) was only 0.48 g P.O. before PCI and 4 g P.O. before and after PCI. This short course of treatment may affect the statistical significance of the results. Furthermore, the other two studies (Wongcharoen et al., 2011; Wongcharoen et al., 2012) adopted a longer course of treatment, a larger total amount of drugs, and a longer follow-up time. The results of the two latter studies showed that curcumin might has cardioprotection in term of anti-inflammation, cardiac function, incidence of in-hospital myocardial infarction and MACE within 30 days after CABG or PCI. The reason why human studies showed drastic difference might be relative to the dosage of curcumin and duration of treatment.

### 4.2 Safety and dose-response of curcumin

According to the existing research results, we found that poor resorption of curcumin was one of the reasons why curcumin was limited clinically. For example, volunteers took a large dose of curcumin 12 g a day in human pharmacokinetic trials. However, the blood concentration was too low to be detected, indicating that its bioavailability was low (Peng and Qian, 2014). Therefore, it is meaningful to find the relationship between the dosage and effectiveness within the safe dosage range of curcumin. First, we conducted a dose-response meta-analysis of animal studies in which curcumin was administered orally. In our model (animal studies), 200 mg/kg BW per day was the optimal dose of curcumin (when dosage ranges from 10 to 200 mg/kg BW per day) and had the best predictive protective effect. This analysis result is consistent with another curcumin dose response analysis result (Lin et al., 2020).

According to other curcumin toxicity studies, 200 mg/kg BW per day is acceptable. Many animal studies reported that significant toxicity, pathological effects, genotoxicity and death were not observed when the maximum intake dose of curcumin reached 5,000 mg/kg BW per day within 14 consecutive days (Wahlström and Blennow, 1978; Perkins et al., 2002; Aggarwal et al., 2016; Damarla et al., 2018). In human studies, when treated with purified nano curcumin, the most remarkable side effects observed include gastrointestinal flatulence (Hanai et al., 2006) and changes in liver function (Na et al., 2013; Zingg et al., 2017). Overall, based on the current research results, curcumin will be safe when it reaches the concentration we predicted.

### 4.3 Potential mechanisms of curcumin in myocardial I/R injury

**FIGURE 12:**
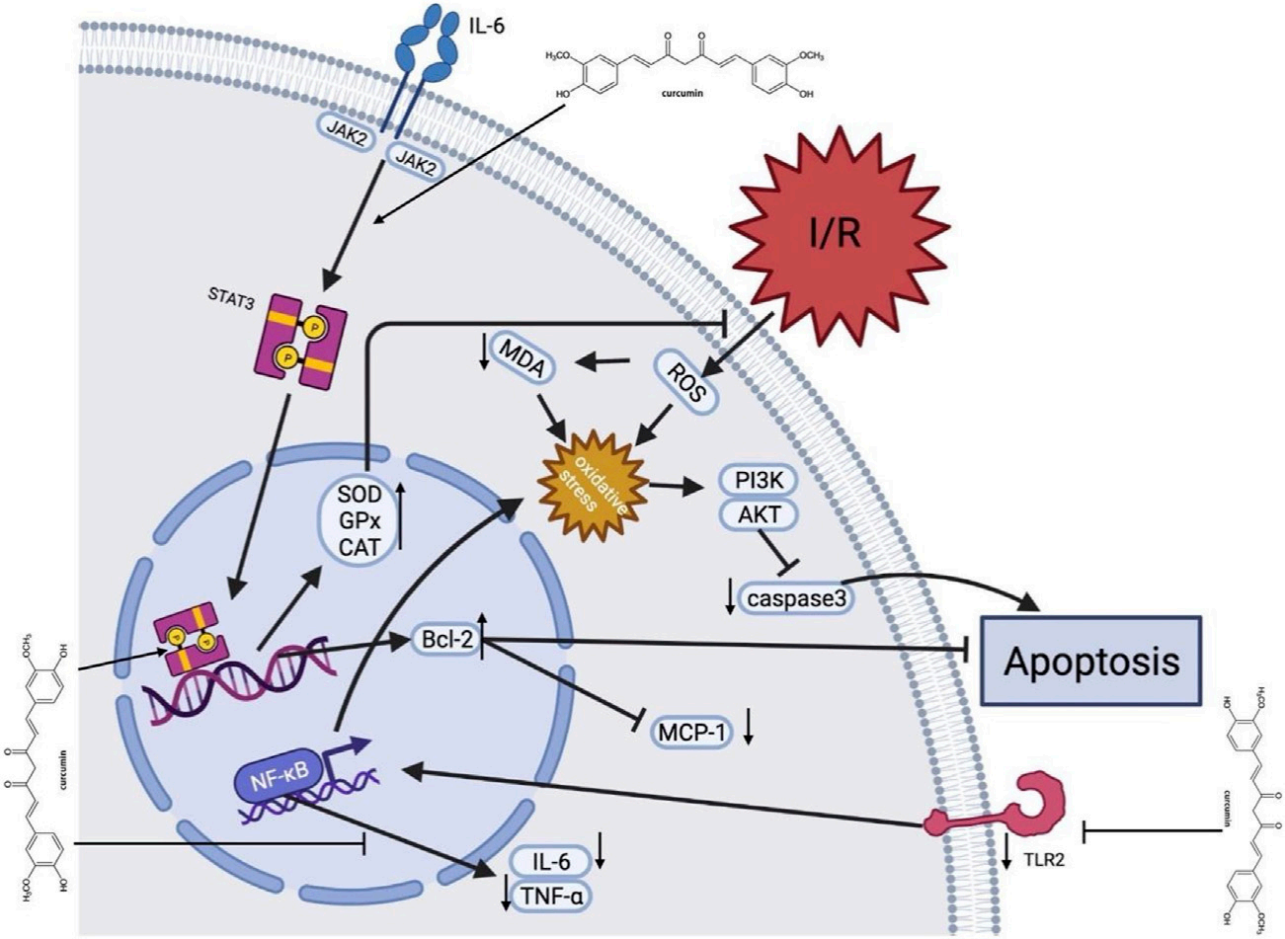
A schematic representation of cardioprotective mechanisms of curcumin for myocardial ischemia/reperfusion injury. After I/R occurs, curcumin inhibits cell apoptosis by activating JAK2/STAT3 pathway and relieving oxidative stress, with upregulated SOD, GPx and CAT expression, decrease of ROS production, and upregulated Bcl-2. In addition, curcumin inhibits the TLR2 and NF-κB pathways on the cell membrane surface *via* downregulation of TNF-α and IL-6. Moreover, curcumin suppresses caspase-3 expression to inhibit apoptosis. AKT, protein kinase B; Bcl-2, B-cell lymphona-2; CAT, catalase; GPx, Glutathione Peroxidase; I/R, ischemia/reperfusion; IL-6, interleukin-6; JAK2, Janus kinase two; McP-1, Monocyte chemotactic protein one; MDA, malondialdehyde; MPO, myeloperoxidase; PI3K, Phosphatidylinositide 3-kinases; ROS, Reactive oxygen species; Superoxide dismutase; STAT3, signal and activator of transcription three; TLR2, Toll-like receptor two; TNF-α, tumor necrosis factor α.

Myocardial I/R injury is associated with various pathophysiological processes, such as calcium overload, generation of oxygen free radicals, endothelial dysfunction, inflammatory response, mitochondrial dysfunction, myocardial cell apoptosis, and autophagy (Kloner et al., 1989; Hausenloy and Yellon, 2003; Yellon and Hausenloy, 2007; Oerlemans et al., 2013; Merz et al., 2019). Considerable evidence indicates that curcumin exerts cardioprotective effects in myocardial I/R injury through multiple molecular mechanisms. Multiple molecular mechanisms might be involved in the underlying protective mechanisms of curcumin against myocardial injury, and a better understanding of these protective mechanisms will provide better insight into curcumin (Figure 12).

#### 4.3.1 Anti-oxidant effect

Oxidative stress plays an essential role in myocardial I/R injury. Reducing oxidative stress is one of the strategies for dealing with I/R injury. The antioxidant activity of curcumin is mainly reflected by scavenging various reactive oxygen species (Broskova et al., 2013). Curcumin could regulate mitochondrial dysfunction after I/R through activating the silent Information regulator 1 (SIRT1) signal, up-regulating Bcl-2, and down-regulating Bax (Mokhtari-Zaer et al., 2018). Some studies also showed a direct antioxidant effect of curcumin, including increasing mitochondrial SOD activity and decreasing the generation of mitochondrial hydrogen peroxide and MDA (Yang et al., 2013a).

#### 4.3.2 Anti-inflammatory effect

Inflammation is usually an active defense mechanism, but excessive inflammation will exacerbate myocardial reperfusion injury. Studies have shown that curcumin exerts anti-inflammatory effects by regulating NF-κB signaling pathway, an essential proinflammatory signaling pathway involved in cardiac injury (Fiorillo et al., 2008). It was shown that curcumin pretreatment over a week reduced TLR2 and MCP1 expression in cardiomyocytes, macrophage infiltration (CD68) and cardiac fibrosis (Kim et al., 2012). In addition, curcumin could inhibit the expression of early growth response 1 (EGR-1) in ischemic myocardial cells through lowering the levels of TNF-α and IL-6 (Wang et al., 2012). This might suggest that curcumin has a potent anti-inflammatory effect and could protect the cardiomyocytes through modulating the expression or activity of proinflammatory cytokines.

#### 4.3.3 Anti-apoptosis effect

Apoptosis is a physiological phenomenon in most circumstances. However, excessive apoptosis under pathological conditions lead to unnecessary cell death. Due to myocardial I/R, STAT3 upregulates the expression of Bcl-2, Bcl-xl, and other genes, which have been shown to reduce cell death and lessen adverse cardiac remodeling after myocardial infarction (Yang et al., 2013b). We found that activation of Janus kinase signal sensor and transcriptional activator (JAK2-STAT3) signal pathway, upregulation of Bcl-2 and downregulation of caspase-3 could be the mechanisms of curcumin in relieving myocardial I/R injury (Duan et al., 2012).

#### 4.3.4 Regulating autophagy effect

Autophagy is functional at low baseline levels in myocardium, removing unnecessary proteins and damaged organelles as a self-protective mechanism (Klionsky et al., 2021). Dysregulated or excessive autophagy could induce cell death (Zhao et al., 2021). A few studies indicated that curcumin might protect mouse cardiomyocytes from oxidative stress by stimulating autophagy (Rainey et al., 2020; Gu et al., 2021; Lin et al., 2021). Other studies claimed that curcumin protects myocardial I/R via downregulating elevated autophagy (Huang et al., 2015; Ma et al., 2021). This suggests that curcumin might be a potential bidirectional regulatory agent in myocardial I/R, either inhibiting excessive autophagy or promoting autophagy. In general, curcumin exerts cardioprotective effect through regulating autophagy.

### 4.4 Implications

Our study found that the animal myocardial IR model is not completely consistent with the characteristics of the clinical patients, and this is because that myocardial ischemia-reperfusion injury (MIR) is often accompanied by a variety of conditions, such as aging, hypertension, diabetes, etc. Therefore, more complex animal models such as aging animal models, should be used to study the pharmacological effects and mechanisms of curcumin in future researches.

The rationality and standardization of in vivo researches directly affect the further transformation from pre-clinical studies to clinical applications. According to SYRCLE 2.0 tool, the methodology quality of included animal studies is moderate. Therefore, when designing experimental protocols, researchers should strictly follow the animal experiment guidelines, such as random allocation, blinding investigators, random outcome assessment, etc., and record this information in the paper.

Many animal studies have shown that curcumin has acceptable security, and no significant toxicity was observed even when rats or mice were given a high dose (5 g/kg BW per day). According to the dose-response model of animal studies, the cardioprotective effect of curcumin may be stronger with the further increase of the dosage. Further, researchers developed a Polylactic-co-glycolic acid (PLGA) and Plga-polyethylene (PEG) (Plga-peg) nanoparticle containing curcumin, and it was observed that the nanoparticles increased the mean half-life of curcumin in about 6 h, enhanced the maximum blood concentration of curcumin by 7.4 times, and improved the bioavailability of curcumin by 55.4 times compared with curcumin aqueous suspension (Khalil et al., 2013). Therefore, we suggest that higher doses of curcumin (more than 200 mg/kg BW per day) and nanoparticle containing curcumin could be explored in future studies to determine the best effective dose.

Conducting more clinical trials should be encouraged because animal experiments have shown excellent effectiveness. In the existing clinical trials, the results showed that curcumin significantly reduced the incidence of MACE in patients after PCI or CABG in the short term. In addition, curcumin is widely consumed as a food material in Southeast Asia, and a large number of studies supported its high biological safety.

In clinical research, researchers should not only focus on the incidence of MACE events caused by MIR in the short term after PCI or CABG, but also focus on the incidence of MACE and cardiac function within 1 year after discharge by increasing follow-up time.

Patients with myocardial injury and reperfusion often need to take antiplatelet drugs. Some studies claimed that curcumin plays an antiplatelet role by inhibiting platelet aggregation (Srivastava et al., 1995; Hirsch et al., 2017), inhibiting cyclooxygenase activity, and blocking calcium channels (Offermanns et al., 1994). However, there is no study explaining the relationship between curcumin and other antiplatelet drugs in vivo experiments.

### 4.5 Limitations

First of all, we only retrieved English and Chinese studies, which may lead to selection bias. Secondly, positive results are no doubt more likely to be published, so the dominance of positive studies may lead to an overestimation of the efficacy of curcumin, although the funnel plot and Egger’s regression test did not show significant publication bias. Thirdly, the overall quality of the animal studies was moderate, ranging from two to six points out of 10, for example, many animal studies ignored the need for randomization. In addition, myocardial infarction usually occurs in patients with cardiovascular risk factors, such as aging, diabetes, hypertension, and hyperlipidemia (Blankstein et al., 2012). However, no animal model with comorbidities was set in the included studies, so their results may be inconsistent with the complexity of the real medical environment.

## 5 Conclusion

Our study is the first study that includes both preclinical and clinical evidence in terms of curcumin in treatment of MIR, and our results suggest that curcumin might play a cardioprotective role in acute myocardial infarction, mainly through its anti-oxidative, anti-inflammatory, anti-apoptosis, and anti-fibrosis effects. In addition, we found that curcumin might need a longer course of treatment and a larger dosage to exert cardioprotection in the clinical studies, and its efficacy is mainly reflected in reducing the incidence of myocardial infarction and MACE in short term. Finally, our study summarized the main defects of the existing research and suggested how such primary studies need to be improved.

## Data Availability

The original contributions presented in the study are included in the article/Supplementary Material, further inquiries can be directed to the corresponding authors.

## Supplementary material

Supplementary Figure S1 Non-linear dose-response relationship between Napierian logarithm of RR and the intake of curcumin. The black lines are regression curves of Ln RR for different curcumin doses. The red line represents the optimal effect. Dots represent each of the studies. RR: ratio of means, equal to meansexperimental/meanscontrol.

## Glossary

AMI: acute myocardial infarction
AST: aspartate aminotransferase
BW: bodyweight
CABG: coronary artery bypass grafting
CAT: catalase
CK: creatine kinase
CK-MB: creatine kinase, MB form
CVD: cardiovascular disease
cTnI: cardiac troponin I
GR: glutathione reductase
GPx: glutathione peroxidase
GST: glutathione S-transferase
GSH: glutathione
hs-CRP: high-sensitive cardiac troponin T
hs-TnT: high-sensitive cardiac troponin T
I/R: Ischemia-reperfusion
IL-6: interleukin-6
LDH: lactate dehydrogenase
LVEF: Left ventricular ejection fraction
LVFS: left ventricular fraction shortening
LVEDd: left ventricular end-diastolic diameter
LVESd: left ventricular end-systolic diameter
MACE: major adverse cardiovascular events
MDA: malonaldehyde
MI: myocardium infarction
MPO: myeloperoxidase
MDA: malondialdehyde
NF-κB: nuclear factor kappa-B
PO: peros
PCI: percutaneous coronary intervention
SOD: superoxide dismutase
TNF-α: tumor necrosis factor alpha.

## References

[B1] Aggarwal M. L. Chacko K. M. Kuruvilla B. T. (2016). Systematic and comprehensive investigation of the toxicity of curcuminoid-essential oil complex: A bioavailable turmeric formulation. Mol. Med. Rep. 13, 592–604. 10.3892/mmr.2015.4579 26648561 PMC4686098

[B2] Ali M. S. Mudagal M. P. Goli D. (2009). Cardioprotective effect of tetrahydrocurcumin and rutin on lipid peroxides and antioxidants in experimentally induced myocardial infarction in rats. Pharmazie 64, 132–136. 19320287

[B3] Aslanabadi N. Entezari-Maleki T. Rezaee H. Jafarzadeh H. R. Vahedpour R. (2019). Curcumin for the prevention of myocardial injury following elective percutaneous coronary intervention; a pilot randomized clinical trial. Eur. J. Pharmacol. 858, 172471. 10.1016/j.ejphar.2019.172471 31228455

[B4] Blankstein R. Ahmed W. Bamberg F. Rogers I. S. Schlett C. L. Nasir K. (2012). Comparison of exercise treadmill testing with cardiac computed tomography angiography among patients presenting to the emergency room with chest pain: The rule out myocardial infarction using computer-assisted tomography (ROMICAT) study. Circ. Cardiovasc Imaging 5, 233–242. 10.1161/CIRCIMAGING.111.969568 22308274

[B5] Broskova Z. Drabikova K. Sotnikova R. Fialova S. Knezl V. (2013). Effect of plant polyphenols on ischemia-reperfusion injury of the isolated rat heart and vessels. Phytother. Res. 27, 1018–1022. 10.1002/ptr.4825 22933407

[B6] Chen X. L. Liu X. R. Fang Y. X. Xu L. (2016). Cardioprotective role of curcumin in myocardial ischemia-reperfusion of male albino rats. Int. J. Clin. Exp. Med. 9.

[B7] Cheng H. Liu W. Ai X. (2005). Protective effect of curcumin on myocardial ischemia reperfusion injury in rats. Zhong Yao Cai 28, 920–922. 16479932

[B8] Cumpston M. Li T. Page M. J. Chandler J. Welch V. A. Higgins J. P. (2019). Updated guidance for trusted systematic reviews: A new edition of the Cochrane handbook for systematic reviews of interventions. Cochrane Database Syst. Rev. 10, ED000142. 10.1002/14651858.ED000142 31643080 PMC10284251

[B9] Damarla S. R. Komma R. Bhatnagar U. Rajesh N. Mulla S. M. A. (2018). An evaluation of the genotoxicity and subchronic oral toxicity of synthetic curcumin. J. Toxicol. 2018, 6872753. 10.1155/2018/6872753 30111997 PMC6077508

[B10] Deng Y. Chen G. Ye M. He Y. Li Z. Wang X. (2018). Bifunctional supramolecular hydrogel alleviates myocardial ischemia/reperfusion injury by inhibiting autophagy and apoptosis. J. Biomed. Nanotechnol. 14, 1458–1470. 10.1166/jbn.2018.2582 29903060

[B11] Duan W. Yang Y. Yan J. Yu S. Liu J. Zhou J. (2012). The effects of curcumin post-treatment against myocardial ischemia and reperfusion by activation of the JAK2/STAT3 signaling pathway. Basic Res. Cardiol. 107, 263. 10.1007/s00395-012-0263-7 22466958

[B12] Fiorillo C. Becatti M. Pensalfini A. Cecchi C. Lanzilao L. Donzelli G. (2008). Curcumin protects cardiac cells against ischemia-reperfusion injury: Effects on oxidative stress, NF-kappaB, and JNK pathways. Free Radic. Biol. Med. 45, 839–846. 10.1016/j.freeradbiomed.2008.06.013 18638545

[B13] Ghosh S. S. Salloum F. N. Abbate A. Krieg R. Sica D. A. Gehr T. W. (2010). Curcumin prevents cardiac remodeling secondary to chronic renal failure through deactivation of hypertrophic signaling in rats. Am. J. Physiol. Heart Circ. Physiol. 299, H975–H984. 10.1152/ajpheart.00154.2010 20601462 PMC2957354

[B14] Gonzalez-Salazar A. Molina-Jijon E. Correa F. Zarco-Marquez G. Calderon-Oliver M. Tapia E. (2011). Curcumin protects from cardiac reperfusion damage by attenuation of oxidant stress and mitochondrial dysfunction. Cardiovasc Toxicol. 11, 357–364. 10.1007/s12012-011-9128-9 21769543

[B15] Gorabi A. M. Hajighasemi S. Kiaie N. Rosano G. M. C. Sathyapalan T. Al-Rasadi K. (2020). Anti-fibrotic effects of curcumin and some of its analogues in the heart. Heart Fail Rev. 25, 731–743. 10.1007/s10741-019-09854-6 31512150

[B16] Grech E. D. Jackson M. J. Ramsdale D. R. (1995). Reperfusion injury after acute myocardial infarction. BMJ 310, 477–478. 10.1136/bmj.310.6978.477 7888877 PMC2548866

[B17] Gu H. P. Wu M. Y. Guo X. H. (2016). Protective effect of curcumin on myocardial ischemia-reperfusion injury in mice and its correlation with Toll-like receptor 4/nuclear factor -κB signaling pathway. Chin. J. Biol. 29, 932–935.

[B18] Gu Y. Xia H. Chen X. Li J. (2021). Curcumin nanoparticles attenuate lipotoxic injury in cardiomyocytes through autophagy and endoplasmic reticulum stress signaling pathways. Front. Pharmacol. 12, 571482. 10.3389/fphar.2021.571482 34456712 PMC8386169

[B19] Hanai H. Iida T. Takeuchi K. Watanabe F. Maruyama Y. Andoh A. (2006). Curcumin maintenance therapy for ulcerative colitis: Randomized, multicenter, double-blind, placebo-controlled trial. Clin. Gastroenterology Hepatology Official Clin. Pract. J. Am. Gastroenterological Assoc. 4, 1502–1506. 10.1016/j.cgh.2006.08.008 17101300

[B20] Hardy N. Viola H. M. Johnstone V. P. Clemons T. D. Cserne Szappanos H. Singh R. (2015). Nanoparticle-mediated dual delivery of an antioxidant and a peptide against the L-Type Ca2+ channel enables simultaneous reduction of cardiac ischemia-reperfusion injury. ACS Nano 9, 279–289. 10.1021/nn5061404 25493575

[B21] Hausenloy D. J. Yellon D. M. (2013). Myocardial ischemia-reperfusion injury: A neglected therapeutic target. J. Clin. Invest. 123, 92–100. 10.1172/JCI62874 23281415 PMC3533275

[B22] Hausenloy D. J. Yellon D. M. (2003). The mitochondrial permeability transition pore: Its fundamental role in mediating cell death during ischaemia and reperfusion. J. Mol. Cell Cardiol. 35, 339–341. 10.1016/s0022-2828(03)00043-9 12689812

[B23] Heusch G. Gersh B. J. (2017). The pathophysiology of acute myocardial infarction and strategies of protection beyond reperfusion: A continual challenge. Eur. Heart J. 38, 774–784. 10.1093/eurheartj/ehw224 27354052

[B24] Hirsch G. E. Viecili P. R. N. de Almeida A. S. Nascimento S. Porto F. G. Otero J. (2017). Natural products with antiplatelet action. Curr. Pharm. Des. 23, 1228–1246. 10.2174/1381612823666161123151611 27881059

[B25] Hooijmans C. R. Rovers M. M. de Vries R. B. Leenaars M. Ritskes-Hoitinga M. Langendam M. W. (2014). SYRCLE's risk of bias tool for animal studies. BMC Med. Res. Methodol. 14, 43. 10.1186/1471-2288-14-43 24667063 PMC4230647

[B26] Huang Z. Ye B. Dai Z. Wu X. Lu Z. Shan P. (2015). Curcumin inhibits autophagy and apoptosis in hypoxia/reoxygenation-induced myocytes. Mol. Med. Rep. 11, 4678–4684. 10.3892/mmr.2015.3322 25673156

[B27] Jeong C. W. Yoo K. Y. Lee S. H. Jeong H. J. Lee C. S. Kim S. J. (2012). Curcumin protects against regional myocardial ischemia/reperfusion injury through activation of RISK/GSK-3β and inhibition of p38 MAPK and JNK. J. Cardiovasc Pharmacol. Ther. 17, 387–394. 10.1177/1074248412438102 22396328

[B28] Jo W. Min B. S. Yang H. Y. Park N. H. Kang K. K. Lee S. (2020). Sappanone A prevents left ventricular dysfunction in a rat myocardial ischemia reperfusion injury model. Int. J. Mol. Sci. 21, 6935. 10.3390/ijms21186935 32967328 PMC7555706

[B29] Khalil N. M. do Nascimento T. C. F. Casa D. M. Dalmolin L. F. de Mattos A. C. Hoss I. (2013). Pharmacokinetics of curcumin-loaded PLGA and PLGA-PEG blend nanoparticles after oral administration in rats. Colloids Surfaces. B, Biointerfaces 101, 353–360. 10.1016/j.colsurfb.2012.06.024 23010041

[B30] Kim Y. S. Kwon J. S. Cho Y. K. Jeong M. H. Cho J. G. Park J. C. (2012). Curcumin reduces the cardiac ischemia-reperfusion injury: Involvement of the toll-like receptor 2 in cardiomyocytes. J. Nutr. Biochem. 23, 1514–1523. 10.1016/j.jnutbio.2011.10.004 22402367

[B31] Kim Y. S. Park H. J. Joo S. Y. Hong M. H. Kim K. H. Hong Y. J. (2008). The protective effect of curcumin on myocardial ischemia-reperfusion injury. Korean Circ. J. 38, 353. 10.4070/kcj.2008.38.7.353

[B32] Klionsky D. J. Petroni G. Amaravadi R. K. Baehrecke E. H. Ballabio A. Boya P. (2021). Autophagy in major human diseases. EMBO J. 40, e108863. 10.15252/embj.2021108863 34459017 PMC8488577

[B33] Kloner R. A. Przyklenk K. Whittaker P. (1989). Deleterious effects of oxygen radicals in ischemia/reperfusion. Resolved and unresolved issues. Circulation 80, 1115–1127. 10.1161/01.cir.80.5.1115 2553296

[B34] Leong P. K. Chen N. Ko K. M. (2013). Schisandrin B enhances hepatic/myocardial glutathione regeneration capacity and protects against oxidant injury in rat livers and hearts. Open Nutraceuticals J. 6, 124–128. 10.2174/1876396001306010124

[B35] Lin K. Chen H. Chen X. Qian J. Huang S. Huang W. (2020). Efficacy of curcumin on aortic atherosclerosis: A systematic review and meta-analysis in mouse studies and insights into possible mechanisms. Oxidative Med. Cell. Longev. 2020, 1520747. 10.1155/2020/1520747 PMC6973199 31998433

[B36] Lin Z. Liu H. Yang C. Zheng H. Zhang Y. Su W. (2021). Curcumin mediates autophagy and apoptosis in granulosa cells: A study of integrated network pharmacology and molecular docking to elucidate toxicological mechanisms. Drug Chem. Toxicol. 45, 2411–2423. 10.1080/01480545.2021.1956941 34315305

[B37] Liu H. Wang C. Qiao Z. Xu Y. (2017). Protective effect of curcumin against myocardium injury in ischemia reperfusion rats. Pharm. Biol. 55, 1144–1148. 10.1080/13880209.2016.1214741 28224816 PMC6130472

[B38] Liu K. Chen H. You Q. S. Ye Q. Wang F. Wang S. (2017). Curcumin attenuates myocardial ischemia-reperfusion injury. Oncotarget 8, 112051–112059. 10.18632/oncotarget.23002 29340110 PMC5762378

[B39] Ma B. Guan G. Lv Q. Yang L. (2021). Curcumin ameliorates palmitic acid-induced saos-2 cell apoptosis via inhibiting oxidative stress and autophagy. Evid. Based Complement. Altern. Med. 2021, 5563660. 10.1155/2021/5563660 PMC8018866 33833814

[B40] Merz S. F. Korste S. Bornemann L. Michel L. Stock P. Squire A. (2019). Contemporaneous 3D characterization of acute and chronic myocardial I/R injury and response. Nat. Commun. 10, 2312. 10.1038/s41467-019-10338-2 31127113 PMC6534576

[B41] Mokhtari-Zaer A. Marefati N. Atkin S. L. Butler A. E. Sahebkar A. (2018). The protective role of curcumin in myocardial ischemia-reperfusion injury. J. Cell Physiol. 234, 214–222. 10.1002/jcp.26848 29968913

[B42] Mozaffarian D. Benjamin E. J. Go A. S. Arnett D. K. Blaha M. J. Cushman M. (2015). Heart disease and stroke statistics--2015 update: A report from the American heart association. Circulation 131, e29–e322. 10.1161/CIR.0000000000000152 25520374

[B43] Na L.-X. Li Y. Pan H.-Z. Zhou X.-L. Sun D.-J. Meng M. (2013). Curcuminoids exert glucose-lowering effect in type 2 diabetes by decreasing serum free fatty acids: A double-blind, placebo-controlled trial. Mol. Nutr. Food Res. 57, 1569–1577. 10.1002/mnfr.201200131 22930403

[B44] Oerlemans M. I. Koudstaal S. Chamuleau S. A. de Kleijn D. P. Doevendans P. A. Sluijter J. P. (2013). Targeting cell death in the reperfused heart: Pharmacological approaches for cardioprotection. Int. J. Cardiol. 165, 410–422. 10.1016/j.ijcard.2012.03.055 22459400

[B45] Offermanns S. Laugwitz K. L. Spicher K. Schultz G. (1994). G proteins of the G12 family are activated via thromboxane A2 and thrombin receptors in human platelets. Proc. Natl. Acad. Sci. U. S. A. 91, 504–508. 10.1073/pnas.91.2.504 8290554 PMC42977

[B46] Orsini N. Li R. Wolk A. Khudyakov P. Spiegelman D. (2012). Meta-analysis for linear and nonlinear dose-response relations: Examples, an evaluation of approximations, and software. Am. J. Epidemiol. 175, 66–73. 10.1093/aje/kwr265 22135359 PMC3244608

[B47] Peng J. R. Qian Z. Y. (2014). Drug delivery systems for overcoming the bioavailability of curcumin: Not only the nanoparticle matters. Nanomedicine (Lond) 9, 747–750. 10.2217/nnm.14.21 24981645

[B48] Perkins S. Verschoyle R. D. Hill K. Parveen I. Threadgill M. D. Sharma R. A. (2002). Chemopreventive efficacy and pharmacokinetics of curcumin in the min/+ mouse, a model of familial adenomatous polyposis. Cancer Epidemiol. Biomarkers Prev. 11, 535–540. 12050094

[B49] Phrommintikul A. Chanchai R. Wongcharoen W. (2019). Effects of curcuminoids on myocardial injury after percutaneous coronary intervention. J. Med. Food 22, 680–684. 10.1089/jmf.2018.4321 31045465

[B50] Rainey N. E. Moustapha A. Petit P. X. (2020). Curcumin, a multifaceted hormetic agent, mediates an intricate crosstalk between mitochondrial turnover, autophagy, and apoptosis. Oxid. Med. Cell Longev. 2020, 3656419. 10.1155/2020/3656419 32765806 PMC7387956

[B51] Ribas N. Garcia-Garcia C. Merono O. Recasens L. Perez-Fernandez S. Bazan V. (2017). Secondary prevention strategies after an acute ST-segment elevation myocardial infarction in the AMI code era: Beyond myocardial mechanical reperfusion. BMC Cardiovasc Disord. 17, 54. 10.1186/s12872-017-0493-6 28173757 PMC5297147

[B52] Saeidinia A. Keihanian F. Butler A. E. Bagheri R. K. Atkin S. L. Sahebkar A. (2018). Curcumin in heart failure: A choice for complementary therapy? Pharmacol. Res. 131, 112–119. 10.1016/j.phrs.2018.03.009 29550354

[B53] Sato M. Cordis G. A. Maulik N. Das D. K. (2000). SAPKs regulation of ischemic preconditioning. Am. J. Physiol. Heart Circ. Physiol. 279, H901–H907. 10.1152/ajpheart.2000.279.3.H901 10993748

[B54] Srivastava K. C. Bordia A. Verma S. K. (1995). Curcumin, a major component of food spice turmeric (Curcuma longa) inhibits aggregation and alters eicosanoid metabolism in human blood platelets. Prostagl. Leukot. Essent. Fat. Acids 52, 223–227. 10.1016/0952-3278(95)90040-3 7784468

[B55] Sterne J. A. C. Savović J. Page M. J. Elbers R. G. Blencowe N. S. Boutron I. (2019). RoB 2: A revised tool for assessing risk of bias in randomised trials. BMJ Clin. Res. ed.) 366, l4898. 10.1136/bmj.l4898 31462531

[B56] Wahlström B. Blennow G. (1978). A study on the fate of curcumin in the rat. Acta Pharmacol. Toxicol. 43, 86–92. 10.1111/j.1600-0773.1978.tb02240.x 696348

[B57] Wang N. P. Pang X. F. Zhang L. H. Tootle S. Harmouche S. Zhao Z. Q. (2014). Attenuation of inflammatory response and reduction in infarct size by postconditioning are associated with downregulation of early growth response 1 during reperfusion in rat heart. Shock 41, 346–354. 10.1097/SHK.0000000000000112 24365880

[B58] Wang N. P. Wang Z. F. Tootle S. Philip T. Zhao Z. Q. (2012). Curcumin promotes cardiac repair and ameliorates cardiac dysfunction following myocardial infarction. Br. J. Pharmacol. 167, 1550–1562. 10.1111/j.1476-5381.2012.02109.x 22823335 PMC3514766

[B59] Wang R. Zhang J. Y. Zhang M. Zhai M. G. Di S. Y. Han Q. H. (2018). Curcumin attenuates IR-induced myocardial injury by activating SIRT3. Eur. Rev. Med. Pharmacol. Sci. 22, 1150–1160. 10.26355/eurrev_201802_14404 29509269

[B60] Wongcharoen W. Jai-Aue S. Phrommintikul A. Nawarawong W. Woragidpoonpol S. Tepsuwan T. (2011). Curcuminoids prevent myocardial infarction after coronary artery bypass grafting. Eur. heart J. 32, 77‐78. 10.1093/eurheartj/ehr322 22481014

[B61] Wongcharoen W. Jai-Aue S. Phrommintikul A. Nawarawong W. Woragidpoonpol S. Tepsuwan T. (2012). Effects of curcuminoids on frequency of acute myocardial infarction after coronary artery bypass grafting. Am. J. Cardiol. 110, 40–44. 10.1016/j.amjcard.2012.02.043 22481014

[B62] Wu C. H. (2011). Protective effect of curcumin on myocardial ischemia-reperfusion injury in rats. Chongqing Med. 40, 25–26.

[B63] Yang Y. Duan W. Jin Z. Yi W. Yan J. Zhang S. (2013). JAK2/STAT3 activation by melatonin attenuates the mitochondrial oxidative damage induced by myocardial ischemia/reperfusion injury. J. Pineal Res. 55, 275–286. 10.1111/jpi.12070 23796350

[B64] Yang Y. Duan W. Lin Y. Yi W. Liang Z. Yan J. (2013). SIRT1 activation by curcumin pretreatment attenuates mitochondrial oxidative damage induced by myocardial ischemia reperfusion injury. Free Radic. Biol. Med. 65, 667–679. 10.1016/j.freeradbiomed.2013.07.007 23880291

[B65] Yao B. H. Jiang W. H. (2014). Curcumin post-treatment has a protective effect on myocardial ischemia/reperfusion injury through heme oxygenase-1. Chin. J. Arteriosclerosis 22, 685–689.

[B66] Yao Q. H. Wang D. Q. Cui C. C. Yuan Z. Y. Chen S. B. Yao X. W. (2004). Curcumin ameliorates left ventricular function in rabbits with pressure overload: Inhibition of the remodeling of the left ventricular collagen network associated with suppression of myocardial tumor necrosis factor-alpha and matrix metalloproteinase-2 expression. Biol. Pharm. Bull. 27, 198–202. 10.1248/bpb.27.198 14758033

[B67] Yeh C. H. Lin Y. M. Wu Y. C. Lin P. J. (2005). Inhibition of NF-kappa B activation can attenuate ischemia/reperfusion-induced contractility impairment via decreasing cardiomyocytic proinflammatory gene up-regulation and matrix metalloproteinase expression. J. Cardiovasc Pharmacol. 45, 301–309. 10.1097/01.fjc.0000155385.41479.b3 15772517

[B68] Yellon D. M. Hausenloy D. J. (2007). Myocardial reperfusion injury. N. Engl. J. Med. 357, 1121–1135. 10.1056/NEJMra071667 17855673

[B69] Zhao Y. G. Codogno P. Zhang H. (2021). Machinery, regulation and pathophysiological implications of autophagosome maturation. Nat. Rev. Mol. Cell Biol. 22, 733–750. 10.1038/s41580-021-00392-4 34302147 PMC8300085

[B70] Zhou X. T. Zou J. J. Ao C. Gong D. Y. Chen X. Ma Y. R. (2020). Renal protective effects of astragaloside IV, in diabetes mellitus kidney damage animal models: A systematic review, meta-analysis. Pharmacol. Res. 160, 105192. 10.1016/j.phrs.2020.105192 32942018

[B71] Zingg J.-M. Hasan S. T. Nakagawa K. Canepa E. Ricciarelli R. Villacorta L. (2017). Modulation of cAMP levels by high-fat diet and curcumin and regulatory effects on CD36/FAT scavenger receptor/fatty acids transporter gene expression. BioFactors Oxf. Engl. 43, 42–53. 10.1002/biof.1307 27355903

