# Supplementary figures and images for: Cardioprotective effects of curcumin against myocardial I/R injury: A systematic review and meta-analysis of preclinical and clinical studies
Source: Front Pharmacol. 2023 Mar 9;14:1111459. doi: 10.3389/fphar.2023.1111459 (PMC10034080; doi:10.3389/fphar.2023.1111459)

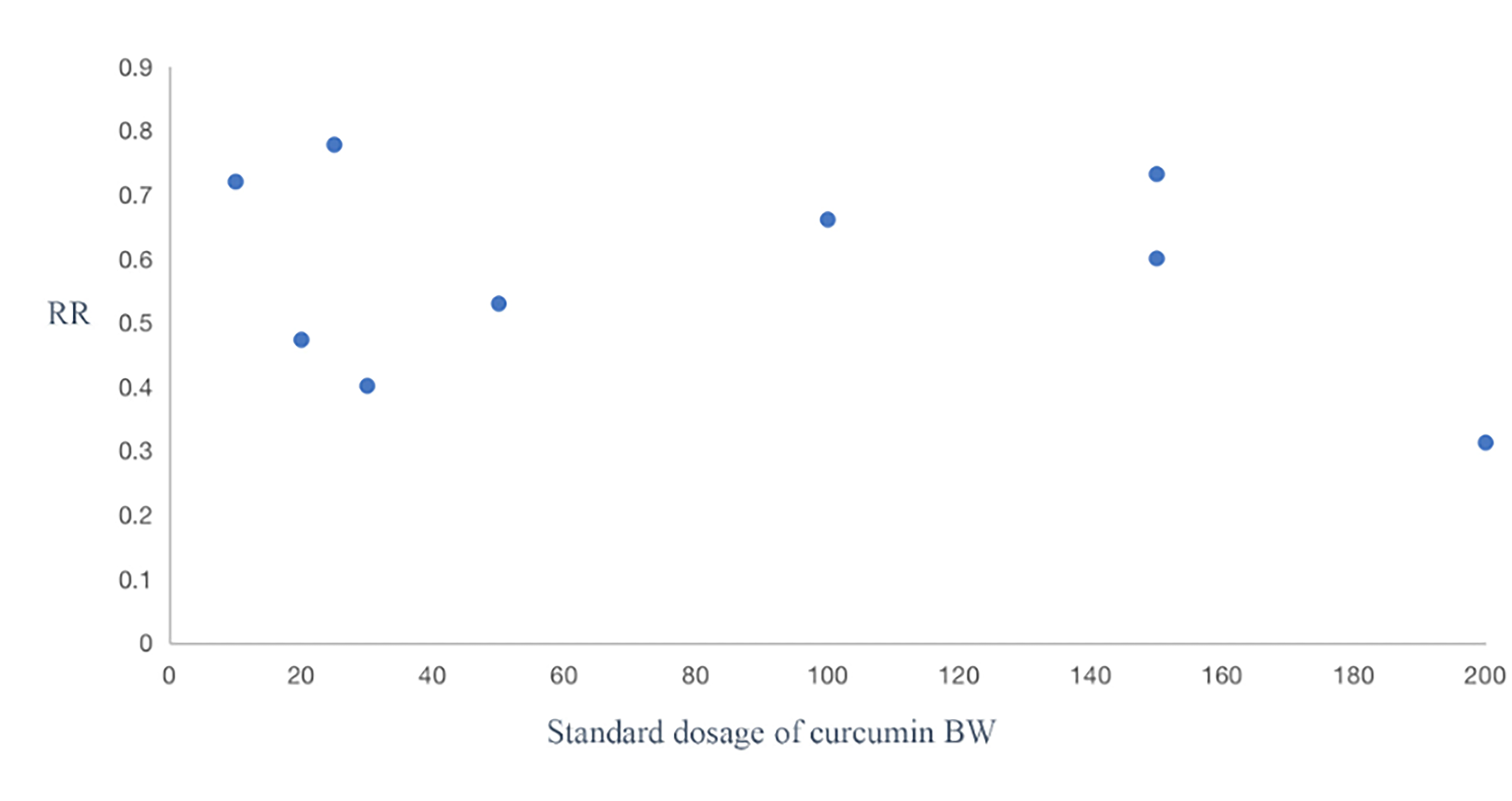

Supplement: Supplementary file 1 [file Image1.TIF]
