# Supplementary material for: Cardioprotective effects of curcumin against myocardial I/R injury: A systematic review and meta-analysis of preclinical and clinical studies
Source: Front Pharmacol. 2023 Mar 9;14:1111459. doi: 10.3389/fphar.2023.1111459 (PMC10034080; doi:10.3389/fphar.2023.1111459)
Supplement: Supplementary file 2 [file DataSheet1.PDF]

Appedix A1: The search strategies of Curcumin for Myocardial Ischemia/Reperfusion Injury

Databases	Search strategies	Hit Counts
**Pubmed (https://pubmed.ncbi.nlm.nih.gov/advanced/)**	#1 ALL fields= “Myocardial Infarction” OR “Myocardial Ischemia” OR “Myocardial Ischemia/Reperfusion Injury” OR “Myocardial I/R” OR “Myocardial I/R Injury” OR “Myocardial Revascularization” OR “Myocardial Reperfusion” OR “Myocardial Stunning” OR “Myocardial Ischemic Preconditioning” OR “Coronary Artery Bypass” OR “Percutaneous Coronary Intervention” OR “Percutaneous Coronary Revascularizations” OR “Percutaneous Transluminal Coronary Intervention”<br>#2 ALL fields= “Turmeric” OR “Curcumin” OR “Curcuma” OR “Curcuminoids” OR “Diferuloylmethane” OR “Desmethoxycurcumin” OR “Bis-desmethoxycurcumin”<br>#3 #1 AND #2	129
**Web of science (https://www.webofscience.com/wos/alldb/advanced-search)**	#1 TS= “Myocardial Infarction” OR “Myocardial Ischemia” OR “Myocardial Ischemia/Reperfusion Injury” OR “Myocardial I/R” OR “Myocardial I/R Injury” OR “Myocardial Revascularization” OR “Myocardial Reperfusion” OR “Myocardial Stunning” OR “Myocardial Ischemic Preconditioning” OR “Coronary Artery Bypass” OR “Percutaneous Coronary Intervention” OR “Percutaneous Coronary Revascularizations” OR “Percutaneous Transluminal Coronary Intervention”<br>#2 TS= “Turmeric” OR “Curcumin” OR “Curcuma” OR “Curcuminoids” OR “Diferuloylmethane” OR “Desmethoxycurcumin” OR “Bis-desmethoxycurcumin”<br>#3 #1 AND #2	191
**Embase (https://www.embase.com)**	#1 ‘Myocardial Infarction’ OR ‘Myocardial Ischemia’ OR ‘Myocardial Ischemia/Reperfusion Injury’ OR ‘Myocardial I/R’ OR ‘Myocardial I/R Injury’ OR ‘Myocardial Revascularization’ OR ‘Myocardial Reperfusion’ OR ‘Myocardial Stunning’ OR ‘Myocardial Ischemic Preconditioning’ OR ‘Coronary Artery Bypass’ OR ‘Percutaneous Coronary Intervention’ OR ‘Percutaneous Coronary Revascularizations’ OR ‘Percutaneous Transluminal Coronary Intervention’<br>#2 ‘Turmeric’ OR ‘Curcumin’ OR ‘Curcuma’ OR ‘Curcuminoids’ OR ‘Diferuloylmethane’ OR ‘Desmethoxycurcumin’ OR ‘Bis-desmethoxycurcumin’<br>#3 #1 AND #2	115
**The Cochrane Library**	#1 ‘Myocardial Infarction’ OR ‘Myocardial Ischemia’ OR ‘Myocardial Ischemia/Reperfusion Injury’ OR ‘Myocardial I/R’ OR ‘Myocardial I/R Injury’ OR ‘Myocardial Revascularization’ OR	7

**(https://www.cochranelibrary.com/advanced-search)**	'Myocardial Reperfusion' OR 'Myocardial Stunning' OR 'Myocardial Ischemic Preconditioning' OR 'Coronary Artery Bypass' OR 'Percutaneous Coronary Intervention' OR 'Percutaneous Coronary Revascularizations' OR 'Percutaneous Transluminal Coronary Intervention'<br>#2 'Turmeric' OR 'Curcumin' OR 'Curcuma' OR 'Curcuminoids' OR 'Diferuloylmethane' OR 'Desmethoxycurcumin' OR 'Bis-desmethoxycurcumin'<br>#3 #1 OR #2	
**CNKI (https://www.cnki.net/)**	(FT=(姜黄+姜黄素) AND FT=(缺血再灌注损伤 + 心肌梗死 + 心肌梗塞 + 心梗 + 再灌注 + 心肌缺血 + 血运重建 + CABG + 冠脉搭桥 + 冠状动脉搭桥 + 冠状动脉旁路移植术 + PCI + 冠脉支架 + 经皮冠状动脉介入术)) AND (SU=(姜黄+姜黄素) OR TKA=(缺血再灌注损伤 + 心肌梗死 + 心肌梗塞 + 心梗 + 再灌注 + 心肌缺血 + 血运重建 + CABG + 冠脉搭桥 + 冠状动脉搭桥 + 冠状动脉旁路移植术 + PCI + 冠脉支架 + 经皮冠状动脉介入术))	37
**WanFang (https://www.wanfangdata.com.cn/)**	全部:(姜黄 or 姜黄素) and 全部:(缺血再灌注损伤 or 心肌梗死 or 心肌梗塞 or 心梗 or 再灌注 or 心肌缺血 or 血运重建 or CABG or 冠脉搭桥 or 冠状动脉搭桥 or 冠状动脉旁路移植术 or PCI or 冠脉支架 or 经皮冠状动脉介入术)	73
**VIP (http://qikan.cqvip.com/index.html)**	U=(姜黄 OR 姜黄素) AND U=(缺血再灌注损伤 OR 心肌梗死 OR 心肌梗塞 OR 心梗 OR 再灌注 OR 心肌缺血 OR 血运重建 OR CABG OR 冠脉搭桥 OR 冠状动脉搭桥 OR 冠状动脉旁路移植术 OR PCI OR 冠脉支架 OR 经皮冠状动脉介入术)	35
**SinoMed (http://www.sinomed.ac.cn/index.jsp)**	("姜黄"[全部字段:智能] OR "姜黄素"[全部字段:智能]) AND("缺血再灌注损伤"[全部字段:智能] OR "心肌梗死"[全部字段:智能] OR "心肌梗塞"[全部字段:智能] OR "心梗"[全部字段:智能] OR "再灌注"[全部字段:智能] OR "心肌缺血"[全部字段:智能] OR "血运重建"[全部字段:智能] OR "CABG "[全部字段:智能] OR "冠脉搭桥"[全部字段:智能] OR "冠状动脉搭桥"[全部字段:智能] OR "冠状动脉旁路移植术"[全部字段:智能] OR "PCI"[全部字段:智能] OR "冠脉支架"[全部字段:智能] OR "经皮冠状动脉介入术"[全部字段:智能])	53
**the Chinese Clinical Trial Registry (http://www.chictr.org.cn/index.as**	在“干预措施”检索框中，依次键入“姜黄”、“姜黄素”。	12

**px)**		
**the World Health Organization International Clinical Trials Registry Platform (https://www. https://clinicaltrials.gov/)**	#1 (title): Turmeric OR Curcumin OR Curcuma OR Curcuminoids OR Diferuloylmethane OR Desmethoxycurcumin OR Bis-desmethoxycurcumin<br>#2 (condition): Myocardial Infarction OR Myocardial Ischemia OR Myocardial Ischemia/Reperfusion Injury OR Myocardial I/R OR Myocardial I/R Injury OR Myocardial Revascularization OR Myocardial Reperfusion OR Myocardial Stunning OR Myocardial Ischemic Preconditioning OR Coronary Artery Bypass OR Percutaneous Coronary Intervention OR Percutaneous Coronary Revascularizations OR Percutaneous Transluminal Coronary Intervention<br>#3 #1 AND #2	2
**ClinicalTrials.gov (www.clinicaltrials.gov/)**	#1 （Intervention/treatment）：Myocardial Infarction OR Myocardial Ischemia OR Myocardial Ischemia/Reperfusion Injury OR Myocardial I/R OR Myocardial I/R Injury OR Myocardial Revascularization OR Myocardial Reperfusion OR Myocardial Stunning OR Myocardial Ischemic Preconditioning OR Coronary Artery Bypass OR Percutaneous Coronary Intervention OR Percutaneous Coronary Revascularizations OR Percutaneous Transluminal Coronary Intervention<br>#2 （Title / Acronym）：Turmeric OR Curcumin OR Curcuma OR Curcuminoids OR Diferuloylmethane OR Desmethoxycurcumin OR Bis-desmethoxycurcumin<br>#3 #1 AND #2	1
